# Micro-computed tomography and histology to explore internal morphology in decapod larvae

**DOI:** 10.1038/s41598-018-32709-3

**Published:** 2018-09-26

**Authors:** Diego Castejón, Javier Alba-Tercedor, Guiomar Rotllant, Enric Ribes, Mercè Durfort, Guillermo Guerao

**Affiliations:** 1 Institut de Ciències del Mar (ICM-CSIC), Passeig Marítim de la Barceloneta 37–49, 08003 Barcelona, Spain; 2 Departamento de Zoología, Facultad de Ciencias, Universidad de Granada, Campus de Fuente Nueva s/n, 18071 Granada, Spain; 3 Unitat de Biologia Cel·lular, Departament de Biologia Cel·lular, Fisiologia i Immunologia, Facultat de Biologia, Universitat de Barcelona, Diagonal 645, 08028 Barcelona, Spain; 4 Independent researcher, 08028 Barcelona, Spain

## Abstract

Traditionally, the internal morphology of crustacean larvae has been studied using destructive techniques such as dissection and microscopy. The present study combines advances in micro-computed tomography (micro-CT) and histology to study the internal morphology of decapod larvae, using the common spider crab (*Maja brachydactyla* Balss, 1922) as a model and resolving the individual limitations of these techniques. The synergy of micro-CT and histology allows the organs to be easily identified, revealing simultaneously the gross morphology (shape, size, and location) and histological organization (tissue arrangement and cell identification). Micro-CT shows mainly the exoskeleton, musculature, digestive and nervous systems, and secondarily the circulatory and respiratory systems, while histology distinguishes several cell types and confirms the organ identity. Micro-CT resolves a discrepancy in the literature regarding the nervous system of crab larvae. The major changes occur in the metamorphosis to the megalopa stage, specifically the formation of the gastric mill, the shortening of the abdominal nerve cord, the curving of the abdomen beneath the cephalothorax, and the development of functional pereiopods, pleopods, and lamellate gills. The combination of micro-CT and histology provides better results than either one alone.

## Introduction

Decapods are an important economic resource that mobilizes billions of dollars per year in fishery and aquaculture production^1^, and decapod larvae play a crucial role in population dynamics, species dispersal, settlement, and recruitment in marine environments^2^. The capacity to explore their internal morphology in detail is important in order to understand these organisms from a functional and evolutionary perspective^3–5^, but few detailed studies have been made on this topic^6–10^. Dissection and histology are the most classical approach, but their invasiveness leads to the alteration and destruction of the specimens^11^. The study of internal morphology received a significant advance with the arrival of confocal laser scanning microscopy (CLSM), which uses stacks of two-dimensional sections to reconstruct three-dimensional models^12,13^. This approach has been applied in non-insect pancrustaceans for the study of embryos and larvae, but it is limited by light penetration and the staining properties of the tissues^14–16^.

A further advance was achieved with the development of micro-computed tomography (micro-CT), which uses the X-ray absorption properties of tissues to obtain the two-dimensional sections required for the reconstruction of a three-dimensional model and provides higher resolution and deeper penetration than CLSM^11,17–20^, allowing to look at the internal morphology of the whole animal without the need of dissection or histology^4^. In adult decapods, this technology has been applied in spiny lobsters to study the ossicles of the stomach^21^, in caridean shrimps to study the alimentary tract^22^, and in brachyuran crabs to describe their general anatomy^23^ and the female reproductive system^24^. It has also been applied to measure the volume of parasitic rhizocephalan structures inside the host body of adult decapods^25^. Recently, Spitzner *et al*.^10^ published a micro-CT–based study focusing on the organogenesis of larvae of the shore crab, *Carcinus maenas*, demonstrating the value of this technique.

Micro-CT is a powerful tool for studying internal morphology but still has some limitations with regard to discriminating adjacent structures with similar X-ray absorbance and resolving the tissue arrangement. Those limitations might be solved by combining micro-CT with histological sections. Hence, the main objective of the present study was to test the combination of micro-CT and histology for studying the general internal morphology of a representative decapod, the common spider crab (*Maja brachydactyla* Balss, 1922), during its larval stages.

## Results

**Figure 1:**
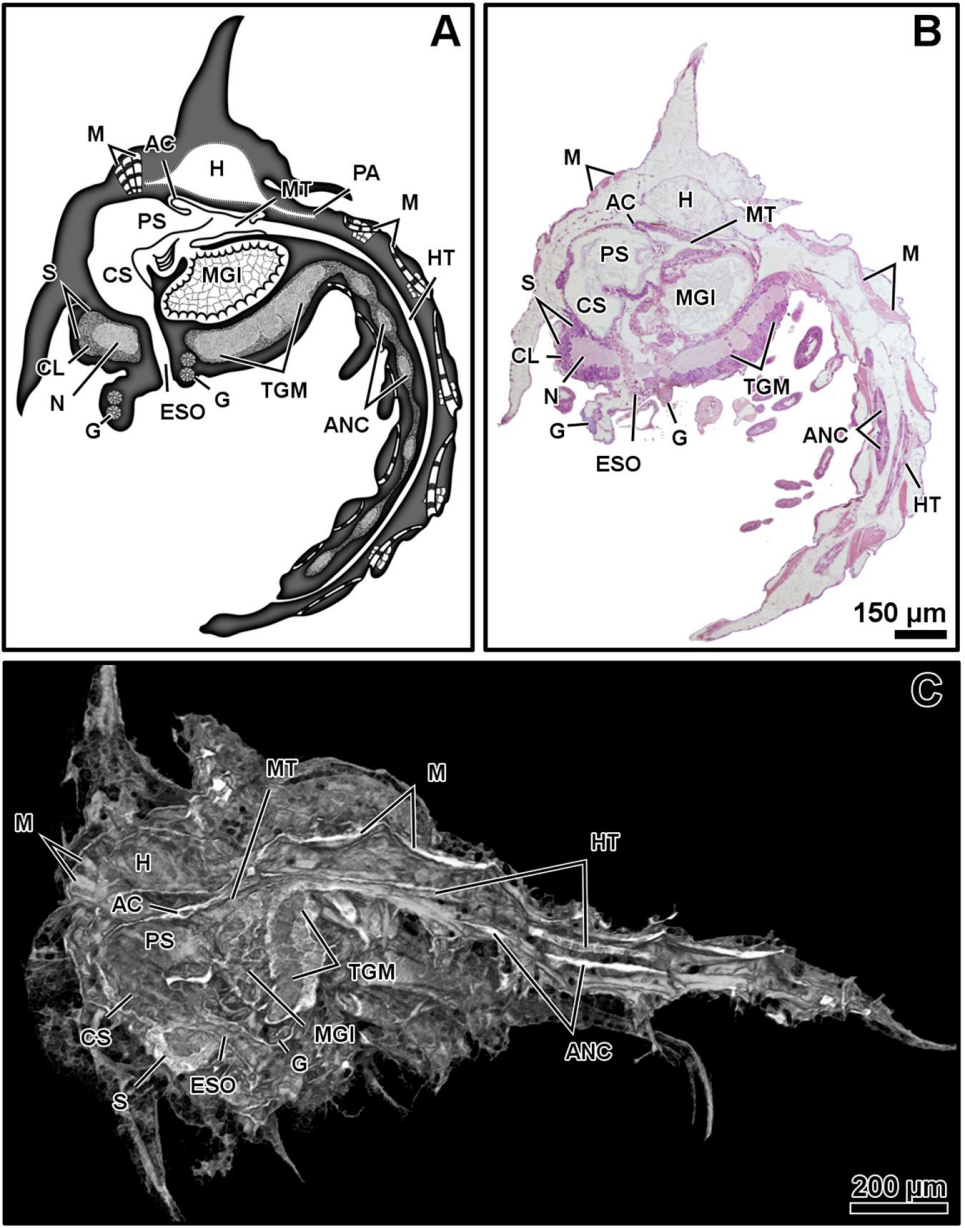
Zoea I, sagittal plane. General diagram (**A**). Histology, haematoxylin-eosin (**B**). Micro-CT volume rendering reconstruction (**C**). Abbreviations: AC, anterior caeca; ANC, abdominal nerve cord; CL, cortical layer of the ganglion; CS, cardiac stomach; ESO, oesophagus; G, glands; H, heart; HT, hindgut tract; M, muscles; MGl, midgut gland; MT, midgut tract; N, neuropile of the ganglion; PA, posterior aorta; PS, pyloric stomach; TGM, thoracic ganglionic mass; S, syncerebrum.

**Figure 2:**
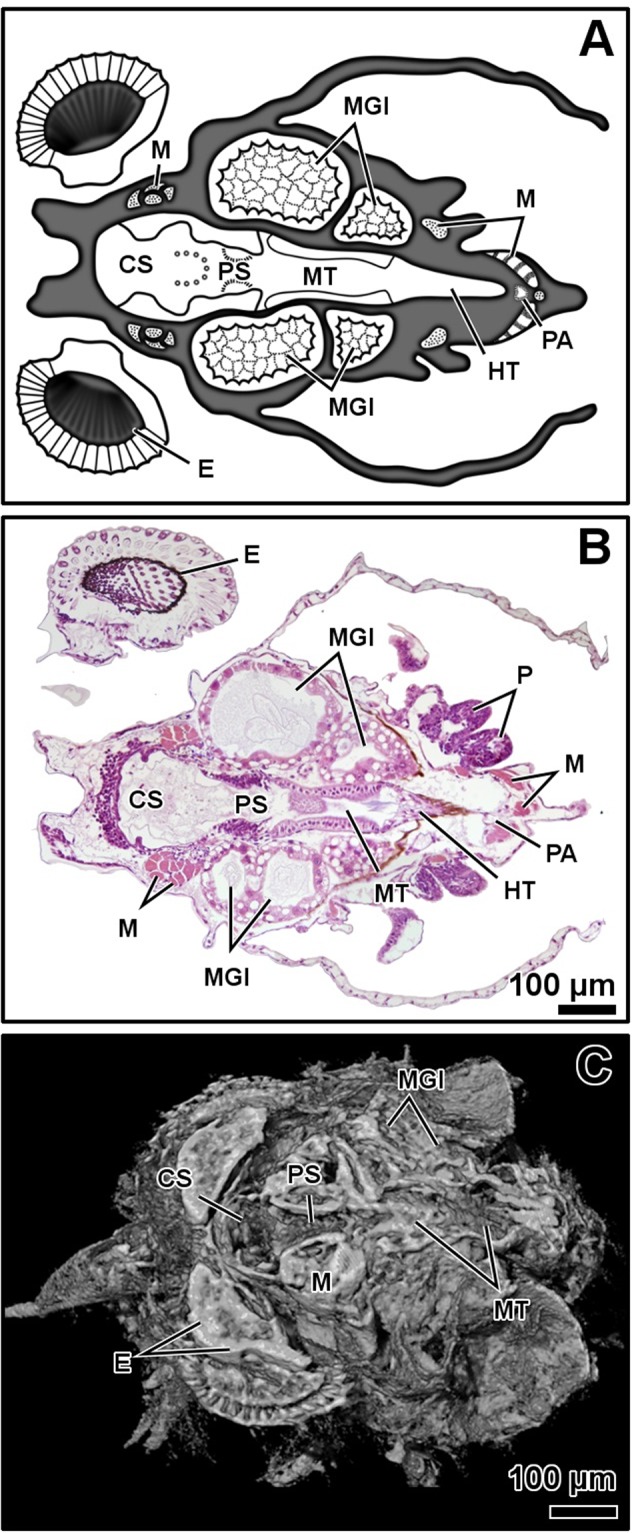
Zoea I, dorso-ventral plane of the digestive system. General diagram (**A**). Histology, haematoxylin-eosin (**B**). Micro-CT volume rendering reconstruction (**C**). Abbreviations: CS, cardiac stomach; E, eye; HT, hindgut tract; M, muscles; MGl, midgut gland; MT, midgut tract; P, pereiopods, PA, posterior aorta; PS, pyloric stomach.

**Figure 3:**
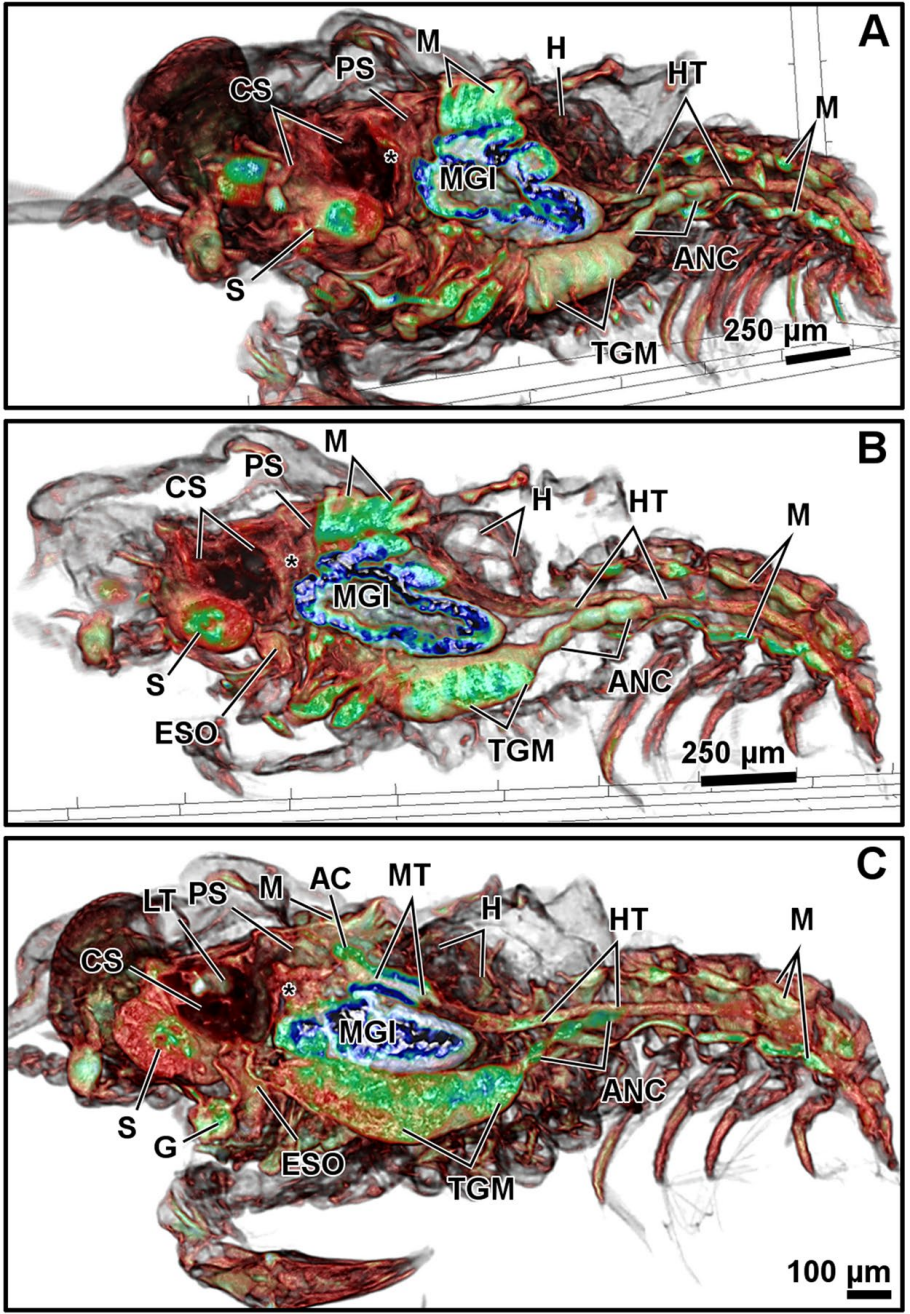
Micro-CT volume rendering reconstructions of different latero-sagittal sections of the megalopa larva from close to the lateral external wall toward the inner-medial body (**A–C**). Abbreviations: (*), cardio-pyloric valve; AC, anterior caeca; ANC, abdominal nerve cord; CS, cardiac stomach; ESO, oesophagus; G, glands; H, heart; HT, hindgut tract; LT, lateral tooth of the gastric mill; M, muscles; MGl, midgut gland; MT, midgut tract; PS, pyloric stomach; TGM, thoracic ganglionic mass; S, syncerebrum.

**Figure 4:**
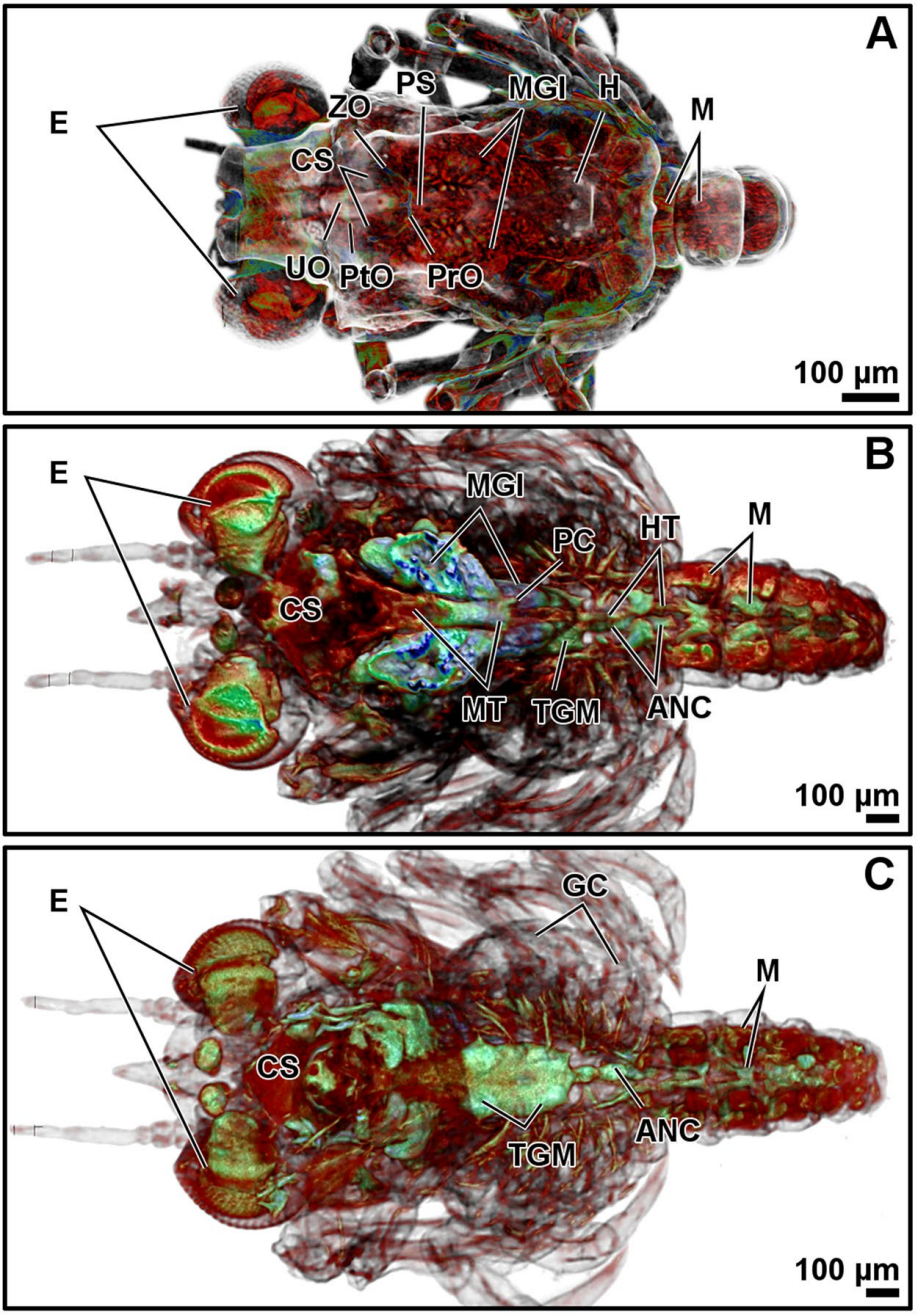
Micro-CT volume rendering reconstructions of the megalopa larva in dorsal (**A**) and ventral (**C**) views. Dorso-ventral plane of the digestive system (**B**). Abbreviations: ANC, abdominal nerve cord; CS, cardiac stomach; E, eyes; GC, gill chamber; H, heart; HT, hindgut tract; M, muscles; MGl, midgut gland; MT, midgut tract; Pro, propyloric ossicle; Pto, pterocardiac ossicle; PC, posterior caecum; PS, pyloric stomach; TGM, thoracic ganglionic mass; UO, urocardiac ossicle; ZO, zygocardiac ossicle.

**Figure 5:**
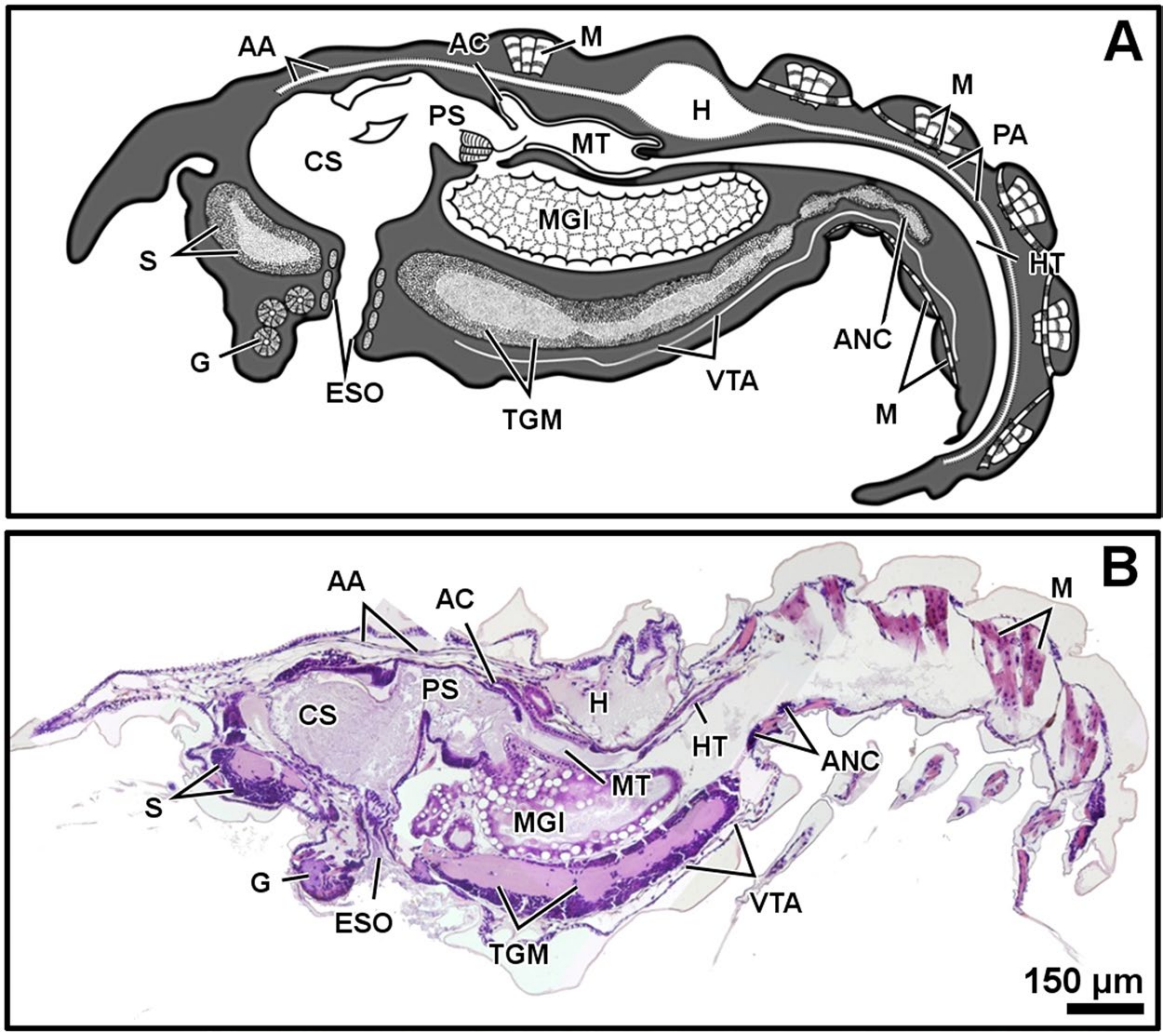
Megalopa, sagittal plane. General diagram (**A**). Histology, haematoxylin-eosin (**B**). Abbreviations: AA, anterior aorta; AC, anterior caeca; ANC, abdominal nerve cord; CS, cardiac stomach; ESO, oesophagus; G, glands; H, heart; HT, hindgut tract; M, muscles; MGl, midgut gland; MT, midgut tract; PA, posterior aorta; PS, pyloric stomach; TGM, thoracic ganglionic mass; S, syncerebrum; VTA, ventral thoracic artery.

**Figure 6:**
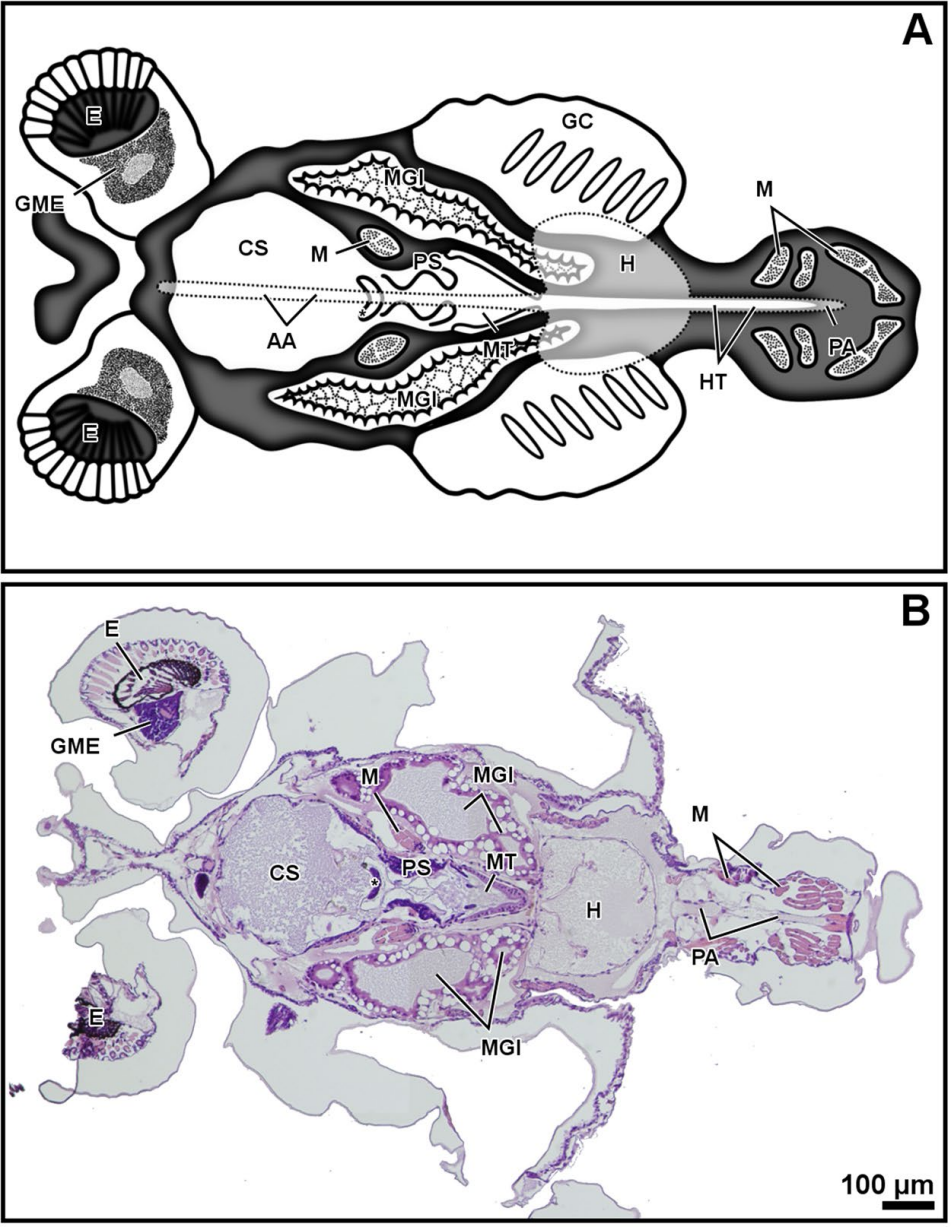
Megalopa, dorso-ventral plane of the digestive system. General diagram (**A**). Histology, haematoxylin-eosin (**B**). Abbreviations: (*), cardio-pyloric valve; AA, anterior aorta; CS, cardiac stomach; GME, ganglionic mass of the eye peduncle; E, eye; GC, gill chamber; H, heart; HT, hindgut tract; M, muscles; MGl, midgut gland; MT, midgut tract; PA, posterior aorta; PS, pyloric stomach.

Micro-CT and histology show the internal organs and other inner structures of the larval stages of *M*. *brachydactyla*, zoea (Figs 1–2) and megalopa (Figs 3–6), resolving their identification and distribution. The internal gross morphology (location and shape of the inner structures) is similar in micro-CT and histology (Figs 1–6). Micro-CT shows better results for studying the shape, volume, and location of several organs, resolving the gross morphology of the anatomy and the relationship among organs (Figs 1–4). The main limitation of micro-CT lies in the difficulty of identifying adjacent structures with similar X-ray absorptive properties. Histology improves the identification of structures with similar X-ray absorptive properties and discerns their tissue arrangement and cellular organization (Figs 1–2 and 5–6).

The resolution of micro-CT discriminates primarily the digestive and nervous systems, the musculature, the exoskeleton, and the eyes, and secondarily the respiratory and circulatory systems (Figs 1–4 and Supplementary Figs (S)8, 11, 13, 15, 17–19 and Videos 1–4). The digestive system is located medially, between the circulatory system (positioned dorsally) and the nervous system (positioned ventrally). It is composed of the oesophagus, stomach, midgut tract, midgut caeca, midgut gland (also known as the hepatopancreas), and hindgut (Figs 1–6 and S1–17 and Videos 1–3). The nervous system is composed of an anterior syncerebrum^26^ (“brain”), the thoracic ganglionic mass, and an abdominal nerve cord formed by a pair of fused nervous chains (Figs 1, 3, 4C and 5 and S1, 3–6, 8, 10, 11, 14, 15, 17 and Videos 1–3). Histology resolves in detail the circulatory and respiratory systems, and reveals the different glands. The circulatory system includes a dorsal heart associated with the main arteries (Figs 1–3, 4A, 5 and 6 and S1, 3–7, 9, 10, 12, 14, 16). The respiratory system increases in complexity after the moult to megalopa, with the formation of gills in the branchial chamber (S14, 15 and 19). The reproductive system is not identified in any larval stage. The elements of the digestive system and nervous system are the easiest organs to observe, so they are described in detail below.

The oesophagus is a short tube that communicates the mouth opening to the ventral floor of the stomach (Figs 1, 3, 5 and S1, 3, 6). In micro-CT, the oesophagus shows a narrowing at the juncture with the stomach (Figs 1C and 3C). Histology reveals a lumen lined by a simple epithelium covered apically by a very thin cuticle (Figs S3, 6) and a continuous band of circular muscles (Figs 1B and S1). The nervous system surrounds the oesophagus (S3) laterally and anteriorly forms a syncerebrum (Figs 1, 3, 5 and S1, 6). Histology also reveals small groups of rosette glands associated with the mouth opening, labrum and mouthparts (Figs 1, 5 and S1, 6), and the antennal glands located on the base of the antennae (S3).

The oesophagus is followed by the stomach; it is subdivided into the cardiac and pyloric stomach by the cardio-pyloric valve (Figs 1–6 and S1–3, 6–13). The stomach of the zoea sustains thick, large setae over the cardio-pyloric valve (S2, 3). The pyloric stomach has postero-ventral pouches protecting the pyloric filters (S10–13). The stomach undergoes a drastic change after the moult to megalopa: the cardiac stomach enlarges antero-laterally and develops the ossicle armature and gastric mill (Figs 3–6 and S6–9). Micro-CT shows the single propyloric and urocardiac ossicles and the paired pterocardiac and zygocardiac ossicles (Fig. 4A), while the sharp teeth of the gastric mill are observed by either micro-CT (Fig. 3C and Video 1 time 0:40-0:45) or histology (S6, 7 and 9). The stomach walls are associated with thick muscle bundles attached to the dorsal tegument (Figs 1–3, 6 and S1–3, 7, 13). Thin muscle bundles connect the cardiac to the pyloric stomach, whose organization is easier to study by histology than by micro-CT (S3). Histology shows a single anterior aorta above the stomach, in addition to a pair of anterolateral arteries crossing between the dorsal muscle bundles (S3, 10, 12).

The midgut tract is a short, cylindrical tube. Both micro-CT and histology show that midgut tract is connected anteriorly to the pyloric stomach, the pair of anterior caeca, and the main tubes of the midgut gland; posteriorly, the midgut tract is connected to the midgut-hindgut junction and the posterior caecum (Figs 1–6 and S1, 2, 6, 8, 13). Histology reveals that the midgut tract has a simple columnar epithelium with a brush border (S1, 2, 4, 6). The midgut tract is associated with three midgut caeca: a pair of anterior caeca and a single posterior caecum. Micro-CT shows the caeca to be short, blind-end tubules with a finger-like shape. Micro-CT discriminates that the pair of anterior caeca are projected forward from the anterior end of the midgut tract; they extend above the pyloric stomach and are partially covered by the adjacent midgut gland and musculature (Figs 3C, 5B and S7, 8, 12, 13 and Video 4); on the other hand, the single posterior caecum projects laterally from the midgut side of the midgut-hindgut junction over the hindgut tract walls (Figs 4B and S8, 13). The pericardium and heart are located above the midgut tract and associated caeca (Figs 1, 3, 5, 6 and S1, 4, 6). Histology shows that the pericardium is a sac-like structure that surrounds the heart intimately with a single layer of plain cells, and that the heart walls are lined by a thin layer of plain cells (S1, 6, 7, 9). Micro-CT and histology reveal that the heart has numerous internal muscular fibres forming trabeculae-like structures (Fig. 3A–C and S7).

The midgut gland (MGl) is the most voluminous organ of the digestive system, and is well shown by micro-CT. It is composed of blind-end tubules forming a pair of symmetric halves laterally covering the pyloric stomach and midgut tract (Figs 3, 4B and S8, 13). Histology shows that the MGl has a simple columnar epithelium with a brush border composed of different cell types: 1) resorptive cells (R) with medium-sized nucleus, lightly stained cytoplasm, and infranuclear lipid vesicle; 2) fibrillar cells (F) with large nucleus and strongly stained cytoplasm; and 3) blister-like cells (B) with a giant supranuclear vesicle (usually called vacuole^27^) (S1, 2, 10, 12, 14). Underlying the midgut tract and the MGl, the thoracic ganglionic mass is observed (Figs 1, 3, 4C, 5 and S1, 4, 6, 8, 10, 11, 14, 15). Its three-dimensional organization is one of the most visible structures in micro-CT. The thoracic ganglionic mass is expanded laterally, acquiring a plate-like shape from which fibre-like nerves extend towards the pereiopods (Fig. 4C).

The hindgut includes the hindgut tract and a thickened anus (S17). Micro-CT reveals the entire length of the hindgut tract as a long tube extending from the midgut-hindgut junction to the last pleonite, whose diameter is smaller than that of the midgut tract (Figs 3B,C, 4B and S8, 13). Histology shows that the lumen of the hindgut is folded and lined by a simple epithelium covered apically by a thin cuticle (S5, 16). Above the hindgut, a single posterior aorta follows the midline of the pleon (S5, 16). Beneath the hindgut, the abdominal nerve cord is observed. Micro-CT reveals in the zoea the presence of the abdominal nerve cord from the first to the fourth pleonite (Fig. 1B,C), while in the megalopa it is displaced anteriorly, compressed, and composed of 3-4 pairs of fused ganglia reaching the second pleonite (Figs 3, 4C and S8, 17).

The branchial chambers of the megalopa contain lamellate gills. Histology shows the gills to have a single layer of squamous epithelial cells covered apically by a thin cuticle (S14), while micro-CT of the gills shows the phyllobranchiate structure of the brachyurans, with parallel lamellae extending from a central filament (S15, 19).

## Discussion

The synergy of micro-CT and histology for studying inner morphology and tissue arrangement is proved in the present study using a decapod species larva as a model. The internal morphology of the zoea shows slight differences from that of the megalopa (e.g. a simpler stomach and longer abdominal nerve cord), whereas the internal morphology of the megalopa resembles that observed in the adult stage^28^. The general morphology of the digestive system of *Maja brachydactyla* coincides largely with that described in other decapods, including adult^29–31^ and larval stages^6–10^. The oesophagus is a short tube whose function is to swallow the food (for an extensive study, see Castejón *et al*.^32^). In the megalopa stage, the stomach develops a gastric mill as reported in numerous species of true crabs (Brachyura)^33–36^ and other decapods^37,38^: it mills the food before it enters the midgut gland^29–31^. Micro-CT reveals that gastric mill teeth are sharp in the megalopa, as reported by scanning electron microscopy in a previous study on *M*. *brachydactyla*^34^. Micro-CT reveals that the midgut gland has a morphology based on blind-end tubules, while histology shows several epithelial cell types, as occur in adult decapods^27,29–31^. Those cell types might be involved in chemical digestion and nutrient absorption^27,29–31^. As reported in adult brachyurans^28,36^, the midgut tract is a short tube, whose role is associated with peritrophic membrane production and nutrient absorption^29–31^. The epithelium of the midgut tract and midgut gland has a brush border identified as microvilli with secretory and absorptive role^30,31^. Micro-CT resolves the gross morphology of the midgut caeca as very short blind-end tubules, as reported in *C*. *maenas* larvae^10^. It is interesting that brachyurans have very small midgut caeca in the larval stages, because in adults they are very long and coiled, unlike in other decapods (specifically shrimps and crayfishes), which have small, vestigial midgut caeca^39^. However, the role of this organ is unclear. The hindgut tract is a large tube coinciding with previous reports of adult brachyurans^28,36^. This organ participates in the osmoregulation and the formation of the feces^29–31^.

The most characteristic feature of the nervous system is the plate-like shape of the thoracic ganglionic mass. This morphology is typical of brachyurans and has been widely reported, including for larval stages^6,9,40^. By contrast, the morphology of the abdominal nerve cord of the megalopa has been a subject of controversy: it has been reported to be extended along the entire pleon^8^, to be an organ with six ganglia displaced anteriorly^6^, and to a short chain composed of three ganglia^9^. The micro-CT employed in the present study resolved this discrepancy, revealing a short organ composed of three to four pairs of fused ganglia displaced anteriorly, occupying only the anterior pleonites.

The internal trabeculae-like structures found on the heart have also been reported in the heart of adult decapods, forming internal sub-chambers^31,41–43^. The vessels are more visible by histology than by micro-CT, adding value to traditional histology as a complementary tool. The location of the main arteries also coincides with that described in other decapods^41–46^, including larval stages^8,10^. The presence of a fully functional circulatory system reveals that from hatching the larvae require a continuous oxygen and nutrient supply.

The internal organs were observed equally by micro-CT and histology, though different procedures were applied: drying for micro-CT and paraffin infiltration for histology. Previous studies have reported that internal organs are observed equally by micro-CT and descriptive studies in honeybees^47^, and by micro-CT and dissected organs in lepidopterans^48^ and marine worms^11^, thus supporting the value of micro-CT as tool for study the internal morphology. Several biomedical studies have also reported a correlation between the models obtained by micro-CT and the images obtained by X-ray radiographies^49^ and histological and ultra-structural sections^50–52^. Therefore, the methodology presented in this study can be applied in several fields; specially, those focused on animal morphology, including biomedical sciences.

In this study, micro-CT resolves the gross morphology and spatial organization and is especially useful for studying the exoskeleton, the nervous system (ganglionic medullas of the eyestalk, syncerebrum, thoracic ganglionic mass, and abdominal nerve cord), the digestive system (oesophagus, stomach, midgut tract, midgut gland, midgut caeca, and hindgut) and the musculature. These findings coincide with those of previous studies in which micro-CT resolves the exoskeleton and its degree of mineralization during the moulting cycle^53^, the neuroanatomy^4,5,10,54–57^, the digestive organs^4,10,55–58^, and the body musculature^3,4,10,18,55,56^. Spitzner *et al*.^10^ recently studied the ontogeny of anatomical structures in *C. maenas* using micro-CT and histology. These authors mainly used micro-CT to resolve the nervous system, the digestive organs, and the body musculature through a segmentation procedure. In the present study, the images were rendered using CTVox and enhanced by transparency, offering additional advantages: the structures have a smoother silhouette and greater detail, and are shown all together, allowing the associations among organ systems to be studied in a more natural context. Therefore, we recommend the CTVox procedure as a complementary tool for resolving animal anatomy. Spitzner *et al*.^10^ employed histological methods to highlight the organs and structures that were not resolved by micro-CT. In the present study we used a different approach: the histological slices were correlated with micro-CT sections to study the same body part and their anatomical structures with both techniques. Our approach allows a direct comparison between techniques and histology resolves the identity and tissue arrangement of the structures observed by micro-CT. Therefore, the combination of micro-CT and histology to study correlated sections allows all organs to be identified easily, revealing simultaneously the gross morphology (shape, size, and location) and histological organization (tissue arrangement and cell identification). The synergy of micro-CT and histology allows histological sectioning to be extrapolated to a third dimension, and provides an optimal set of tools for describing the internal morphology of small animals.

## Methods

### Animal culture

The species selected as a model was the common spider crab (*Maja brachydactyla* Balss, 1922), which has two larval phases: the zoea and the megalopa^59^. The larvae were obtained and cultured using a method successfully applied in a previous study^60^. In brief, adult specimens (six females and one male) were maintained in 2000-L cylindrical tanks at a renewal rate of 3.5 m^3^ h^−1^, 18 ± 1 °C, a salinity of 35 ± 1 ppt, and 12 light hours per day, and were fed with mussels. The larvae within ca. 15 hours after hatch (zoeae) were collected from the adult tanks and cultured in 600-mL glass beakers placed inside incubation chambers at a temperature of 21 ± 1 °C, a salinity of 35 ± 1 ppt, and 12 light hours per day, and were fed with *Artemia* sp. nauplii.

### Histology

Around 30 zoeae and megalopae were examined. The whole larvae were fixed in Davidson’s fixative (absolute ethanol, filtered seawater, 37% formaldehyde, glycine and glacial acetic acid in a proportion of 3:3:2:1:1) for 24 h. Then, they were dehydrated in an ethanol and xylene series (70% ethanol for 3 h, then 96% ethanol, 100% ethanol, 100% ethanol, a 1:1 solution of ethanol and xylene, 100% xylene, and 100% xylene; 1 h per bath) and infiltrated with paraffin (2 baths, 6 h each) using an automatic tissue processor (Myr, Spain). The paraffin blocks were prepared in a paraffin processor composed of a dispensing module for heated liquid paraffin and a table-top cooling plate as a cooling module (Myr, Spain). The blocks were sliced into 2-µm sections using a microtome (Leica RM2155, Leica Microsystems, Wetzlar, Germany). Previously to staining, the paraffin was removed from the slices with 100% xylene (2 baths, 10 min per bath) and slices were rehydrated (100% ethanol, 90% ethanol, 70% ethanol, and distillate water; 5 min each bath). Then, they were stained with haematoxylin (Haematoxylin Solution Harris Modified, HHS32-1L Merck former Sigma-Aldrich, Germany) and eosin (Eosin Y Solution, HT110232-1L Merck former Sigma-Aldrich, Germany), following the protocol: 1) staining with haematoxylin for 5-6 min, 2) cleaning with tap water for 5-10 min, and 3) staining with eosin for 4-5 min. Finally, the slices were dehydrated (96% ethanol, 100% ethanol, 100% ethanol, 100% xylene, 100% xylene; 5 min each bath) and mounted in glass microscope slides (mounting medium: Eukitt® Quick-hardening mounting medium, 03989-100 mL Merck former Sigma-Aldrich, Germany). The observations were performed in optical microscope (Leica LB30T 111/97) connected to a camera (Olympus DP70 1.45 Mpx; Olympus Corporation, Germany) and an image analysing system (DP Controller 2.1.1.83 and DP Manager 2.1.1.163; Olympus Corporation, Germany).

### Micro-computed tomography (micro-CT)

The zoea was fixed in a solution of cacodylate buffer (0.1 mol L^−1^ pH 7.4) with 2% paraformaldehyde and 2.5% glutaraldehyde for 12 h at 4 °C inconstant darkness. Then, the zoea was rinsed twice in the same buffer and post-fixed in cacodylate buffer (0.1 mol L^−1^ pH 7.4) with 1% osmium tetroxide solution. The megalopae were fixed with a solution of 70% ethanol. The larvae were rinsed, dehydrated and preserved in 100% isopropanol and stained with a solution of 1% iodine in absolute ethanol for 72 h. Following previous studies^61^, the larvae were submerged in hexamethyldisilazane for 4-5 hours and air-dried overnight. The dried specimens were placed in holders specially designed for small-sized animals, and the mounting system was selected according to the animal size following Alba-Tercedor and Sáinz-Cantero^62^. A single zoea and two megalopae were mounted and examined. The zoea was mounted on the tip of a nylon filament line (a 200-µm diameter nylon fishing line) and glued using cyanoacrylate. The megalopae were mounted inside a small piece of BASOTECT® (melamine resin foam, BASF Chemical Company), whose very low density gives low absorptive properties for the X-ray, so it can be easily eliminated during the segmentation procedure^47^. All samples were enclosed inside a plastic tube to avoid any movement caused by the forced refrigerating air during the scanning process.

The larvae were scanned in a SkyScan 1172 high-resolution microtomographer (Bruker microCT, Kontich, Belgium) with a Hamamatsu 80/250 source and a VDS 1.3 Mp camera. The scanning parameters for the zoea was setup as follows: isotropic voxel size 1.48 µm per pixel, source voltage 49 kV, source current 78 µA, and image rotation scan 180° with a 0.3° rotation step. The scanning parameters for the megalopae were setup as follows: isotropic voxel size 1.47 µm per pixel, source voltage 54 kV, source current 85 µA, and image rotation scan 180° with a 0.5° rotation step.

Bruker microCT SkyScan (www.skyscan.be) software (NRecon, DataViewer, CTAnalyser) was used for primary reconstructions and for the “cleaning” process to obtain the datasets of cross-sectional images (slices). The volume rendering images were obtained with the free SkyScan software CTVox (colour volume rendering images were obtained by varying the colour transfer function curves, in conjunction with the lighting and shadowing options). For a more detailed description of the process, see Alba-Tercedor^63^. Micro-CT studies were performed at the Department of Zoology of the University of Granada, Spain.

## Electronic supplementary material

Video 1

Video 2

Video 3

Video 4

Supplementary Information and Supplementary Figures
