# Supplementary material for: Micro-computed tomography and histology to explore internal morphology in decapod larvae
Source: Sci Rep. 2018 Sep 26;8:14399. doi: 10.1038/s41598-018-32709-3 (PMC6158171; doi:10.1038/s41598-018-32709-3)

Supplementary Information.

Micro-computed tomography and histology to explore internal morphology in decapod larvae

Diego Castejón, Javier Alba-Tercedor, Guiomar Rotllant, Enric Ribes, Mercè Durfort, and Guillermo Guerao

Abstract

Traditionally, the internal morphology of crustacean larvae has been studied using destructive techniques such as dissection and microscopy. The present study combines advances in micro-computed tomography (micro-CT) and histology to study the internal morphology of decapod larvae, using the common spider crab (*Maja brachydactyla* Balss, 1922) as a model and resolving the individual limitations of these techniques. The synergy of micro-CT and histology allows the organs to be easily identified, revealing simultaneously the gross morphology (shape, size, and location) and histological organization (tissue arrangement and cell identification). Micro-CT shows mainly the exoskeleton, musculature, digestive and nervous systems, and secondarily the circulatory and respiratory systems, while histology distinguishes several cell types and confirms the organ identity. Micro-CT resolves a discrepancy in the literature regarding the nervous system of crab larvae. The major changes occur in the metamorphosis to the megalopa stage, specifically the formation of the gastric mill, the shortening of the abdominal nerve cord, the curving of the abdomen beneath the cephalothorax, and the development of functional pereiopods, pleopods, and lamellate gills. The combination of micro-CT and histology provides better results than either one alone.

Videos. Legends.

Video 1. CTVox's volume rendering video of the spider crab Megalopa larva. The internal organs and structures appear in different colors in accordance with their opacity to X-ray.

Video 2. CTVox's volume rendering video of the spider crab Megalopa larva. The external skeleton has been transparent by software while the internal organs and structures appear in different colors in accordance with their opacity to X-ray.

Video 3. CTVox's volume rendering video of the spider crab Megalopa larva. The external skeleton has been transparent by software while the internal organs and structures appear in different colors in accordance with their opacity to X-ray.

Video 4. CTVox's volume rendering video of the spider crab Megalopa larva. The midgut gland and adjacent musculature has been sectioned to show the pair of anterior midgut caeca. The internal organs and structures appear in different colors in accordance with their opacity to X-ray.

Supplementary Figures. Legends.

Supplementary Figure 1 (S1). *Maja brachydactyla*. Zoea I. Sagittal section at digestive system plane. Haematoxylin-eosin.

Supplementary Figure 2 (S2). *Maja brachydactyla*. Zoea I. Dorso-ventral section at digestive system plane. Haematoxylin-eosin.

Supplementary Figure 3 (S3). *Maja brachydactyla*. Zoea I. Transverse section at cardio-pyloric valve plane. Haematoxylin-eosin.

Supplementary Figure 4 (S4). *Maja brachydactyla*. Zoea I. Transverse section at midgut tract plane. Haematoxylin-eosin.

Supplementary Figure 5 (S5). *Maja brachydactyla*. Zoea I. Transverse section at hindgut tract plane (pleon). Haematoxylin-eosin.

Supplementary Figure 6 (S6). *Maja brachydactyla*. Megalopa. Sagittal section at digestive system plane. Haematoxylin-eosin.

Supplementary Figure 7 (S7). *Maja brachydactyla*. Megalopa. Dorso-ventral section at anterior midgut caeca plane. Haematoxylin-eosin.

Supplementary Figure 8 (S8). *Maja brachydactyla*. Megalopa. Dorso-ventral section at anterior midgut caeca plane. Micro-CT volume rendering reconstruction.

Supplementary Figure 9 (S9). *Maja brachydactyla*. Megalopa. Dorso-ventral section at digestive system plane. Haematoxylin-eosin. Abbreviations: CS, cardiac stomach; H, heart; LT, lateral teeth of the gastric mill; M, muscles; MGl, midgut gland; MT, midgut tract; P, pericardium; PS, pyloric stomach; PA; posterior aorta; T, trabeculae.

Supplementary Figure 10 (S10). *Maja brachydactyla*. Megalopa. Transverse section at pyloric filters plane. Haematoxylin-eosin.

Supplementary Figure 11 (S11). *Maja brachydactyla*. Megalopa. Transverse section at pyloric filters plane. Micro-CT volume rendering reconstruction.

Supplementary Figure 12 (S12). *Maja brachydactyla*. Megalopa. Transverse 45 degrees section showing the pyloric filters and the anterior midgut caeca. Haematoxylin-eosin.

Supplementary Figure 13 (S13). *Maja brachydactyla*. Megalopa. Transverse 45 degrees section showing the pyloric filters and the anterior midgut caeca. Micro-CT volume rendering reconstruction.

Supplementary Figure 14 (S14). *Maja brachydactyla*. Megalopa. Transverse section at midgut tract plane. Haematoxylin-eosin.

Supplementary Figure 15 (S15). *Maja brachydactyla*. Megalopa. Transverse section at midgut tract plane. Micro-CT volume rendering reconstruction.

Supplementary Figure 16 (S16). *Maja brachydactyla*. Megalopa. Transverse section at hindgut tract plane (pleon). Haematoxylin-eosin.

Supplementary Figure 17 (S17). *Maja brachydactyla*. Megalopa. Lateral view showing the pleon and abdominal nerve cord. Micro-CT volume rendering reconstruction.

Supplementary Figure 18 (S18). *Maja brachydactyla*. Megalopa. Lateral view showing the external exoskeleton. Micro-CT volume rendering reconstruction.

Supplementary Figure 19 (S19). *Maja brachydactyla*. Megalopa. Lateral view showing the gills. Micro-CT volume rendering reconstruction.

Supplementary Figure 1

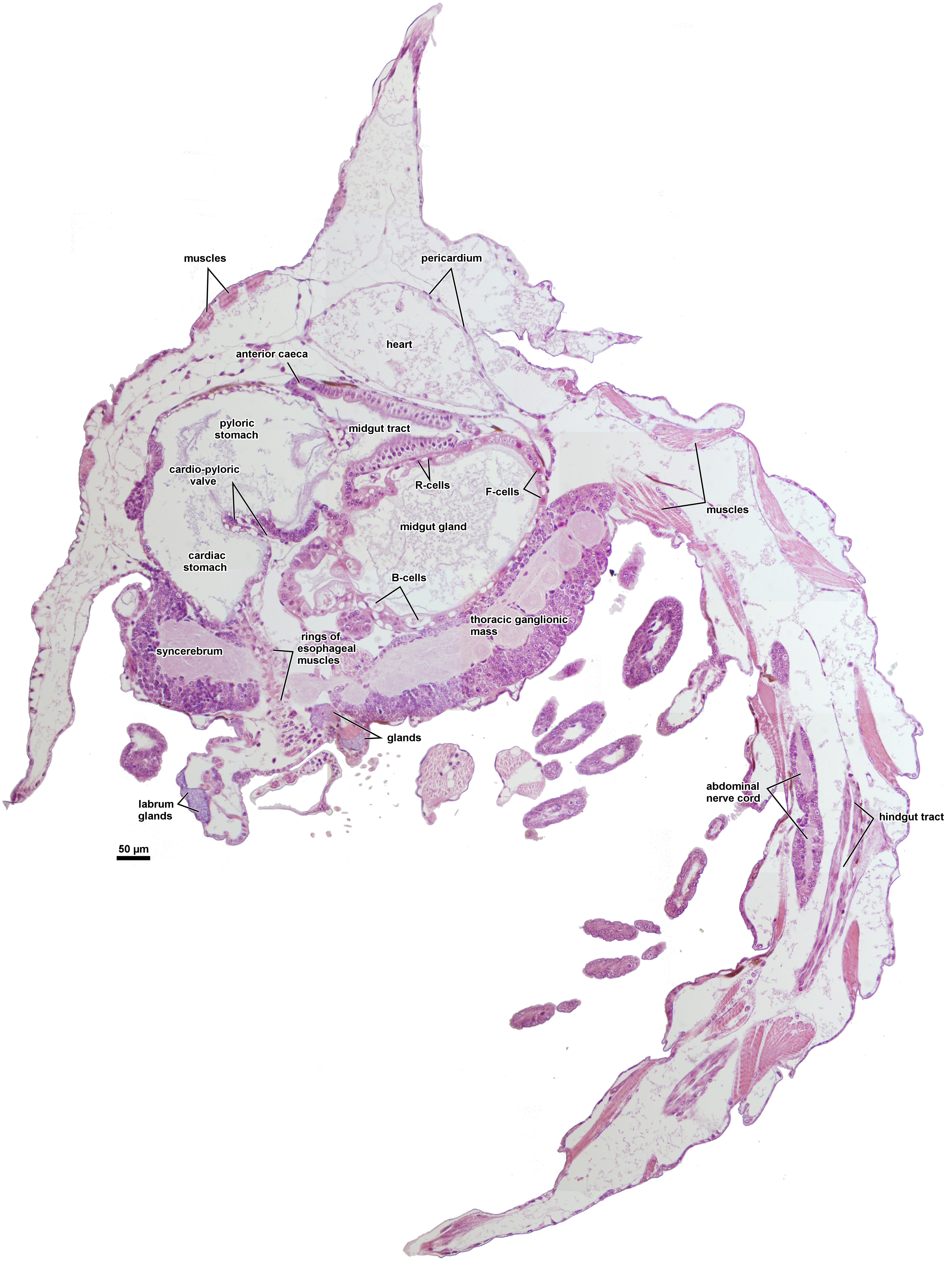

Supplementary Figure 2

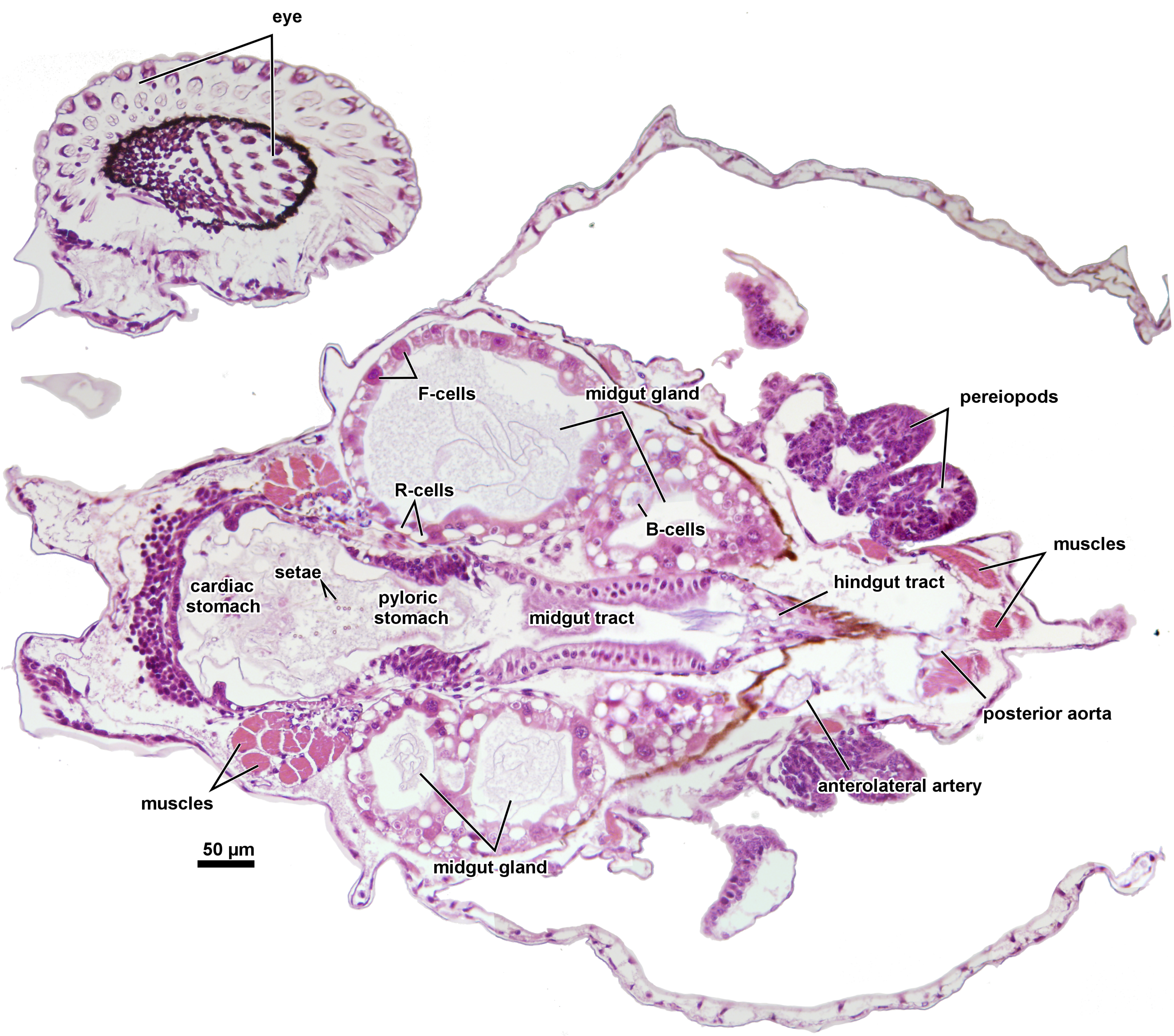

Supplementary Figure 3

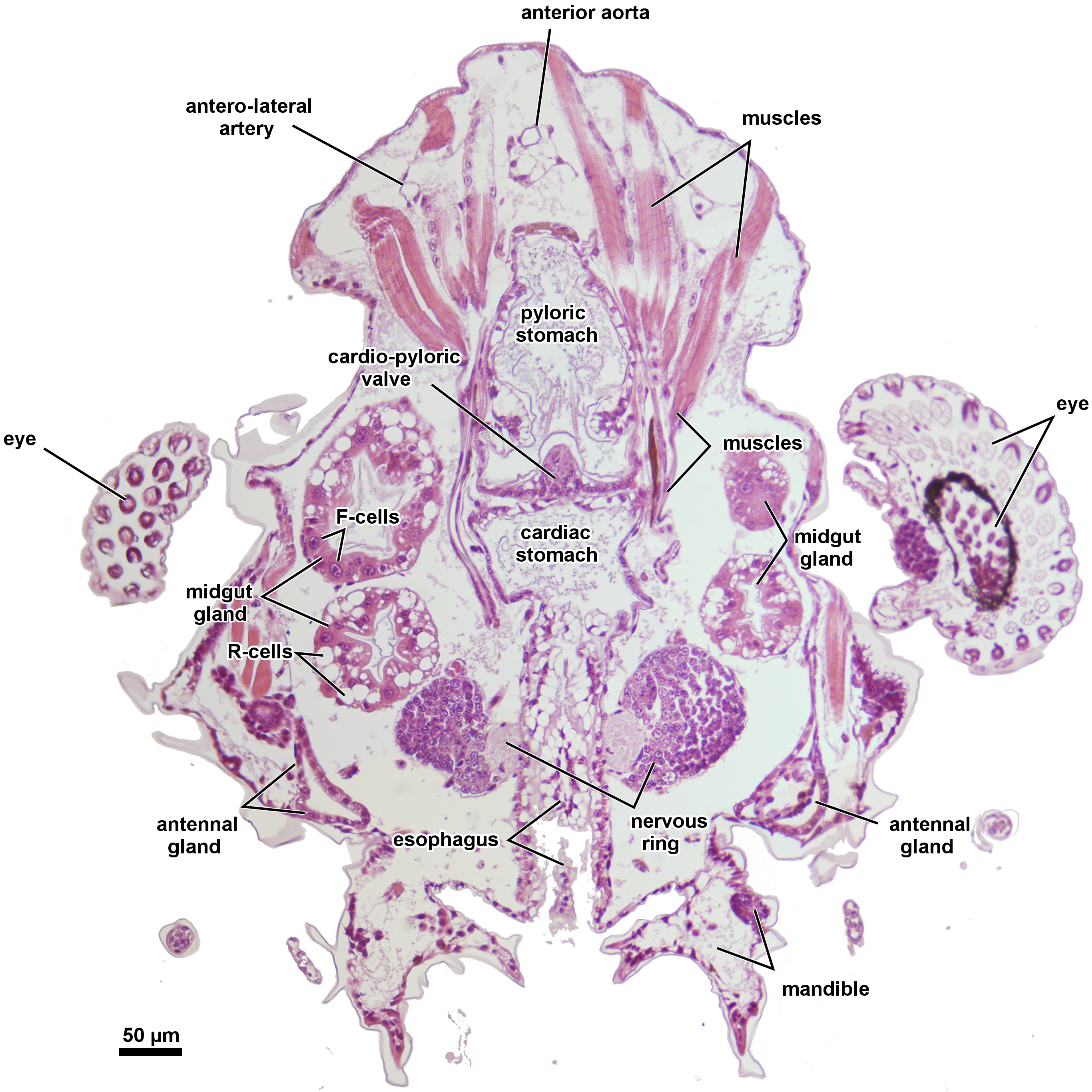

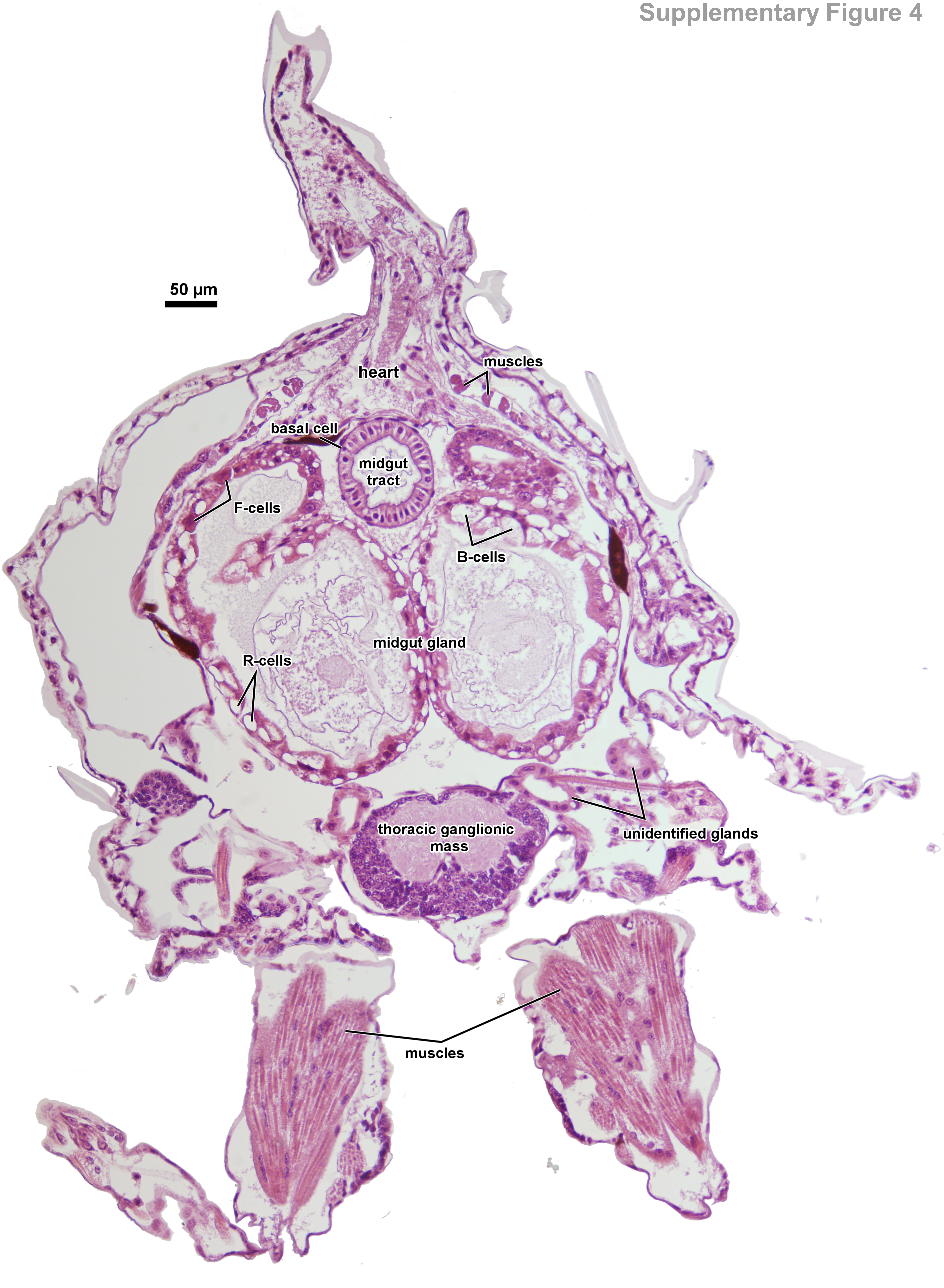
50 µm
heart
muscles
basal cell
midgut
tract
F-cells
B-cells
midgut gland
R-cells
thoracic ganglionic
mass
unidentified glands
muscles

Supplementary Figure 5

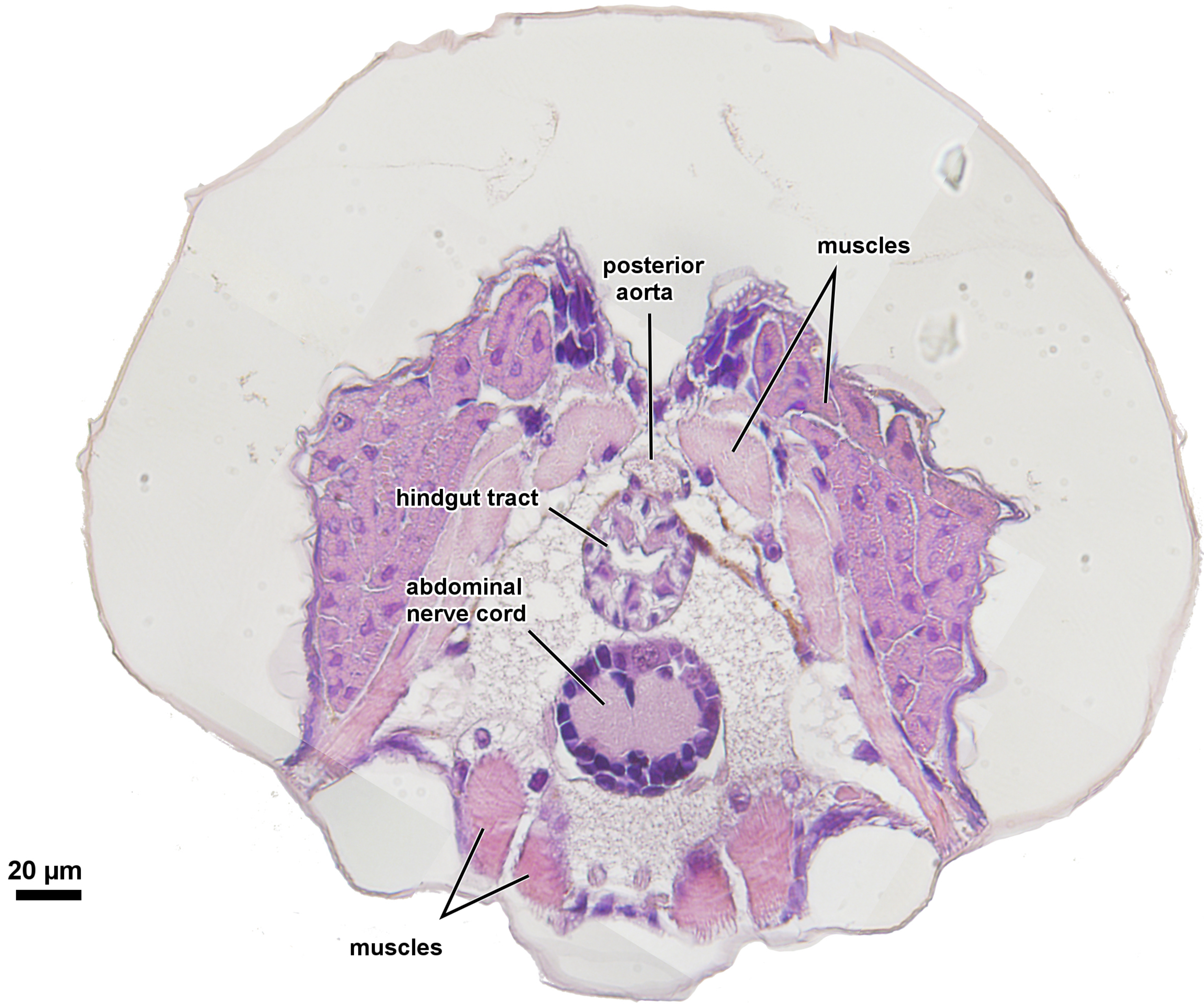

Supplementary Figure 6

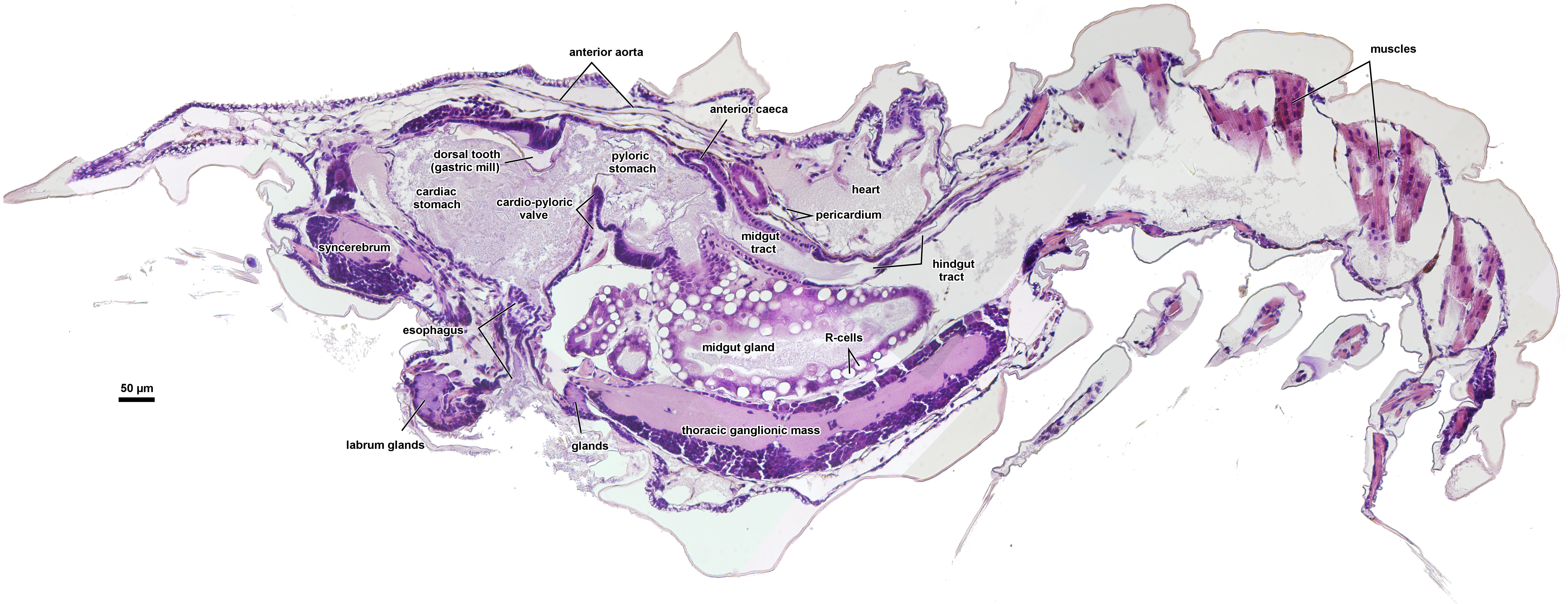

Supplementary Figure 7

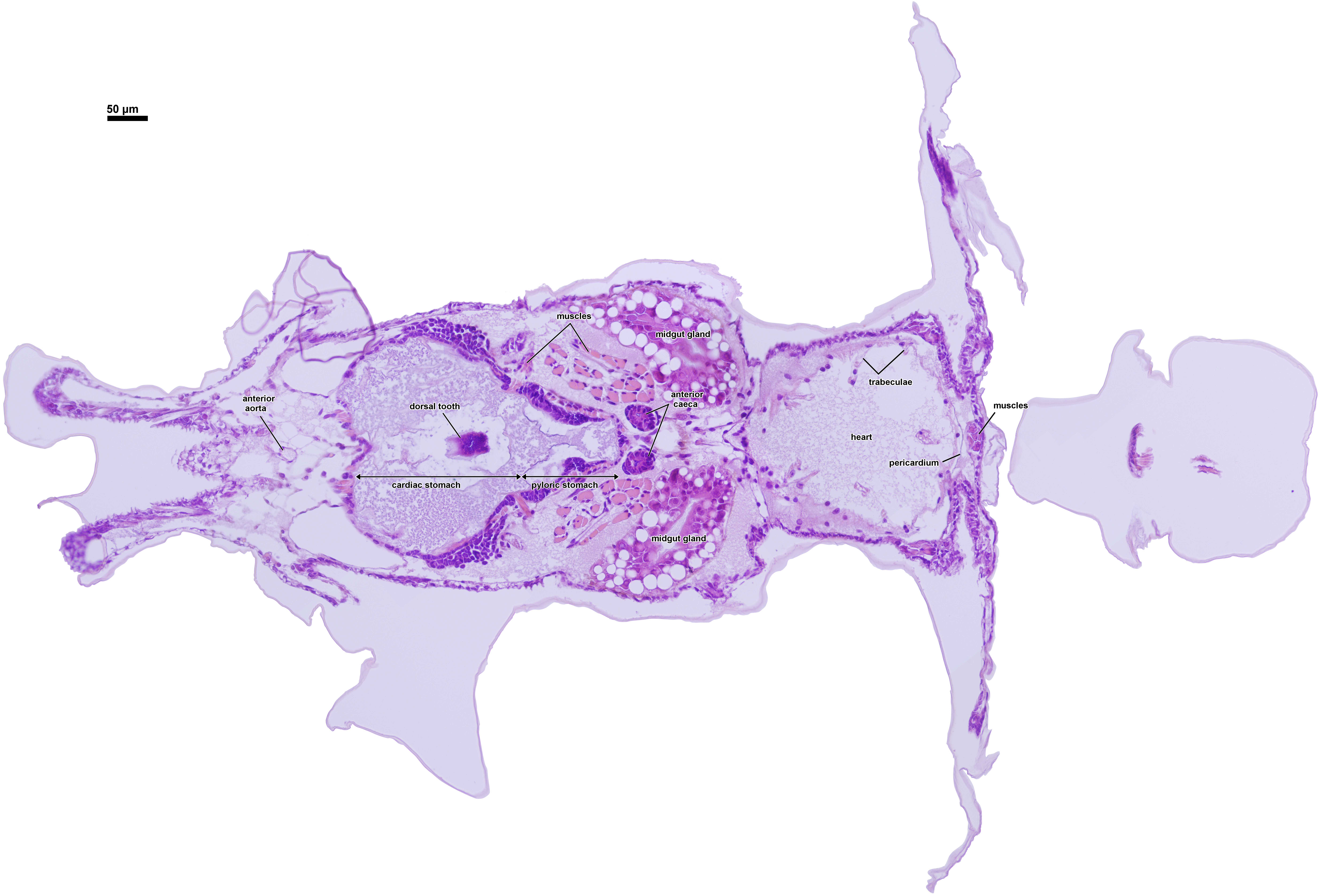

Supplementary Figure 8

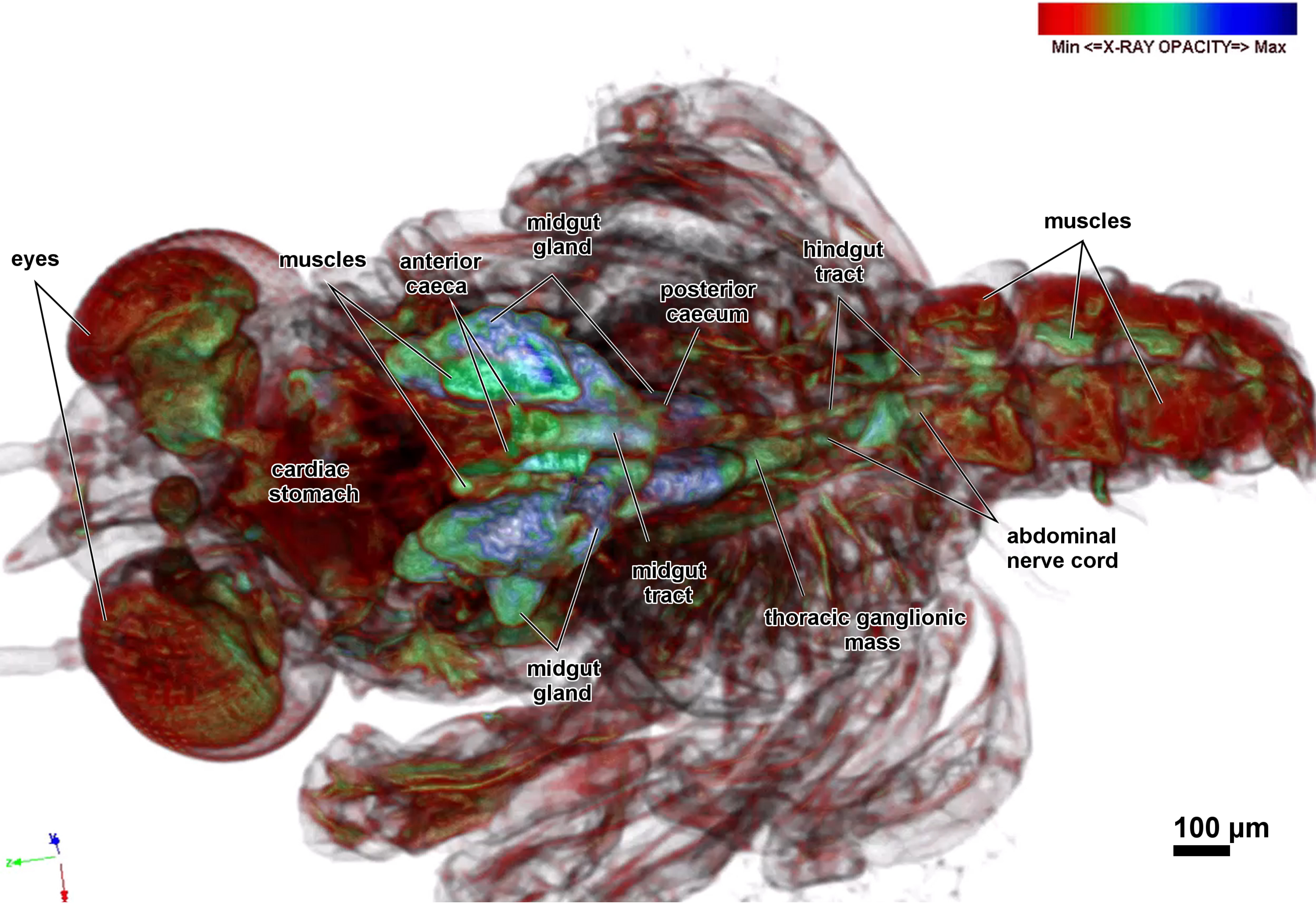

Supplementary Figure 9

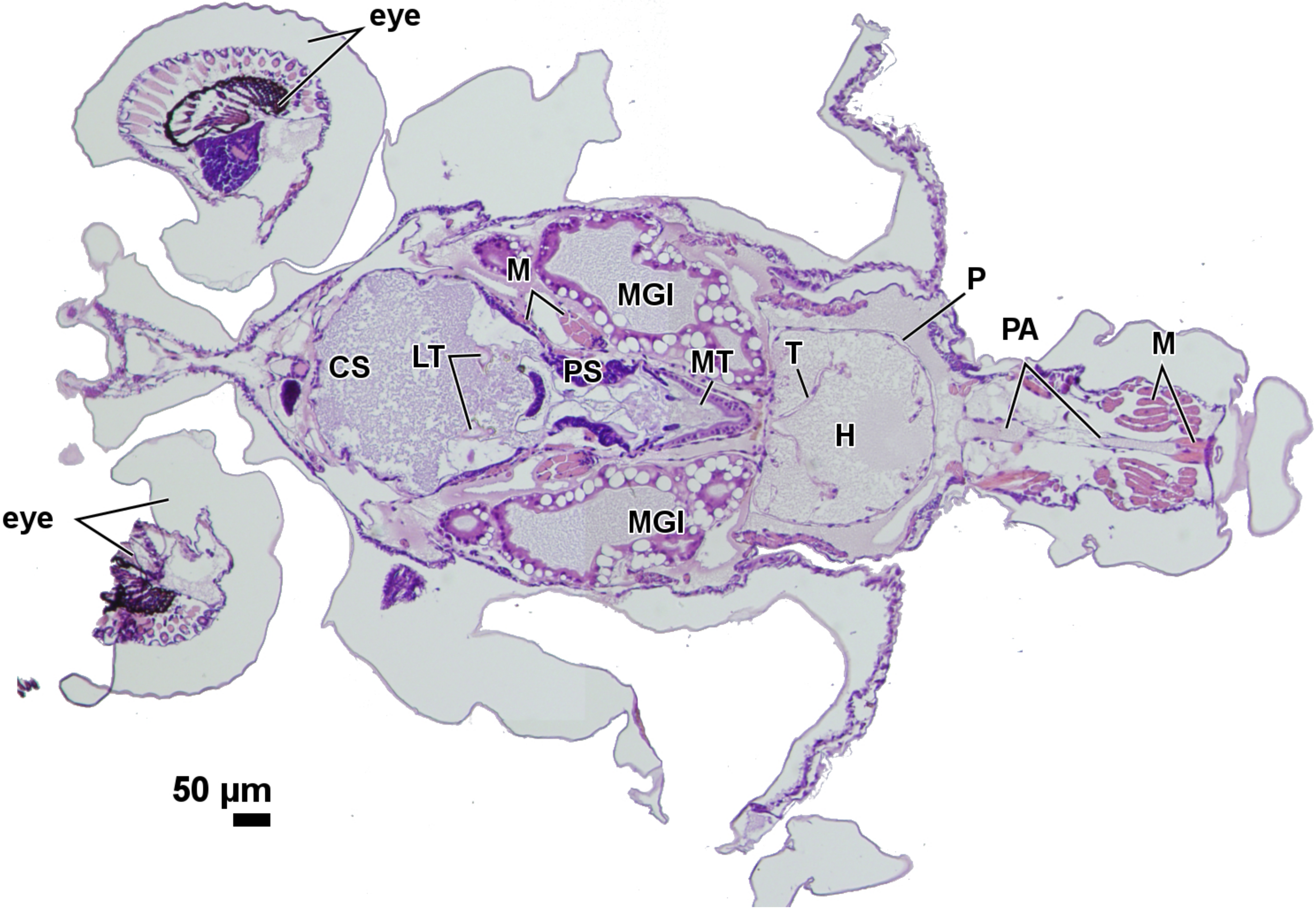

Supplementary Figure 10

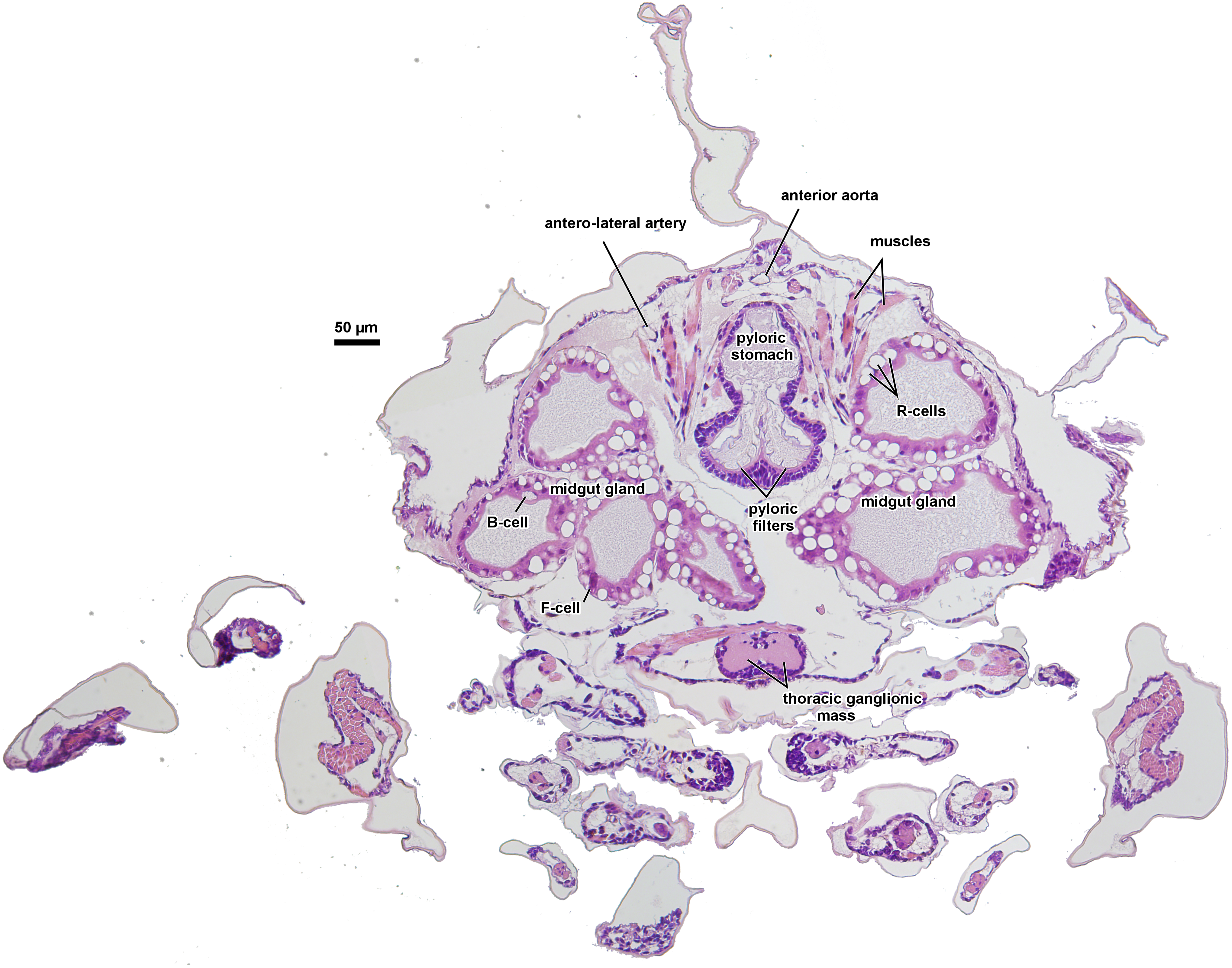

Supplementary Figure 11

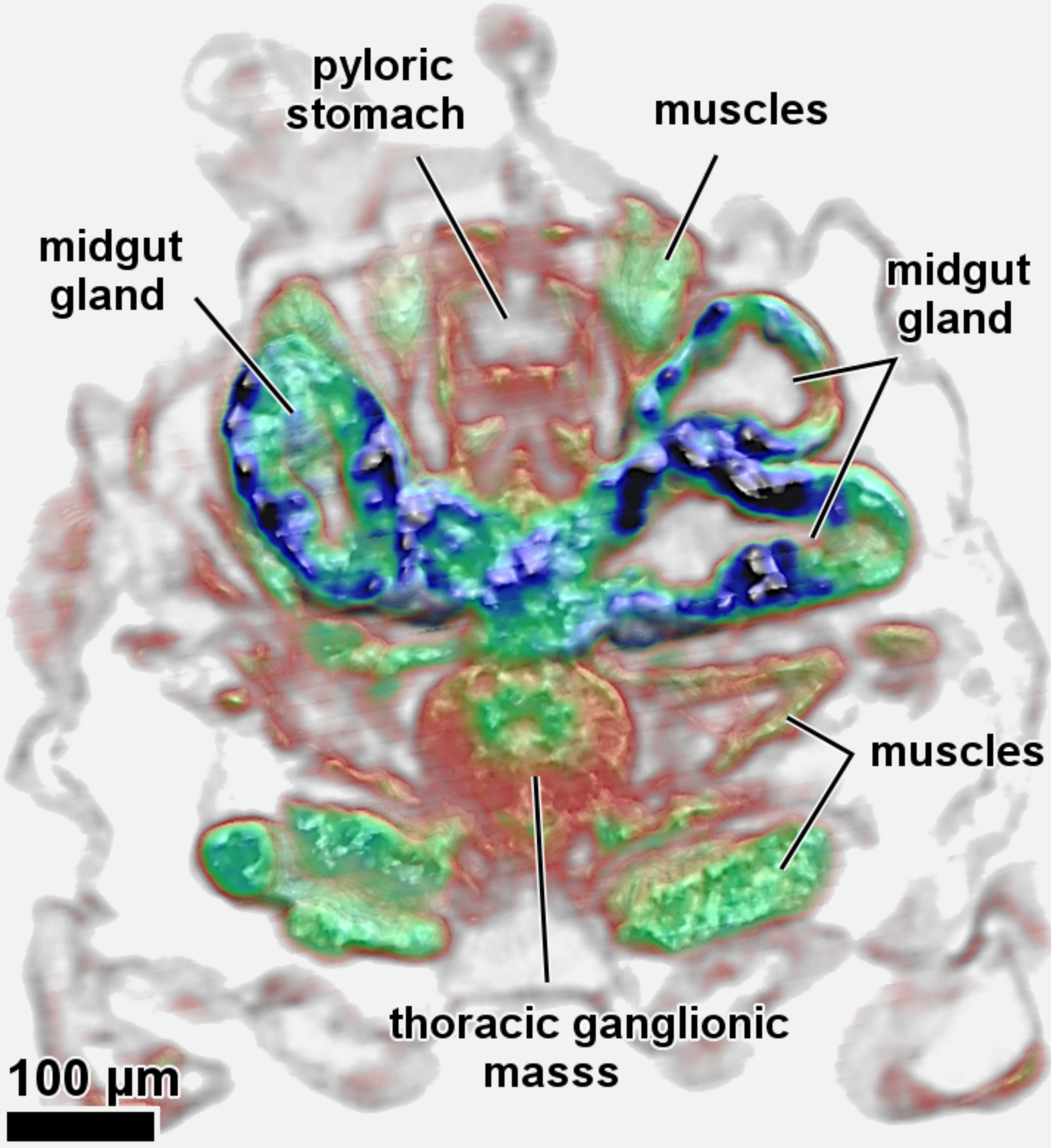

Supplementary Figure 12

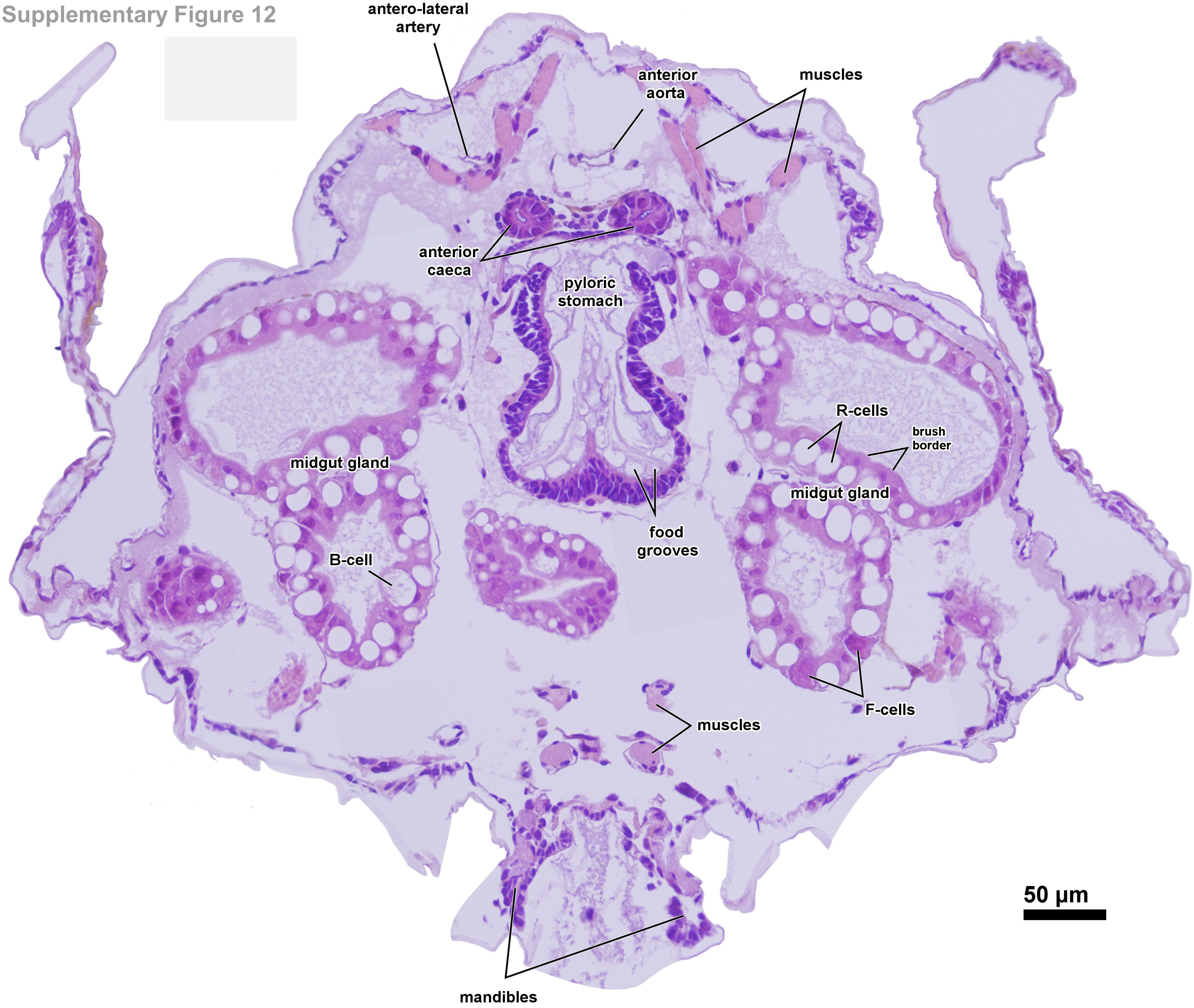

Supplementary Figure 13

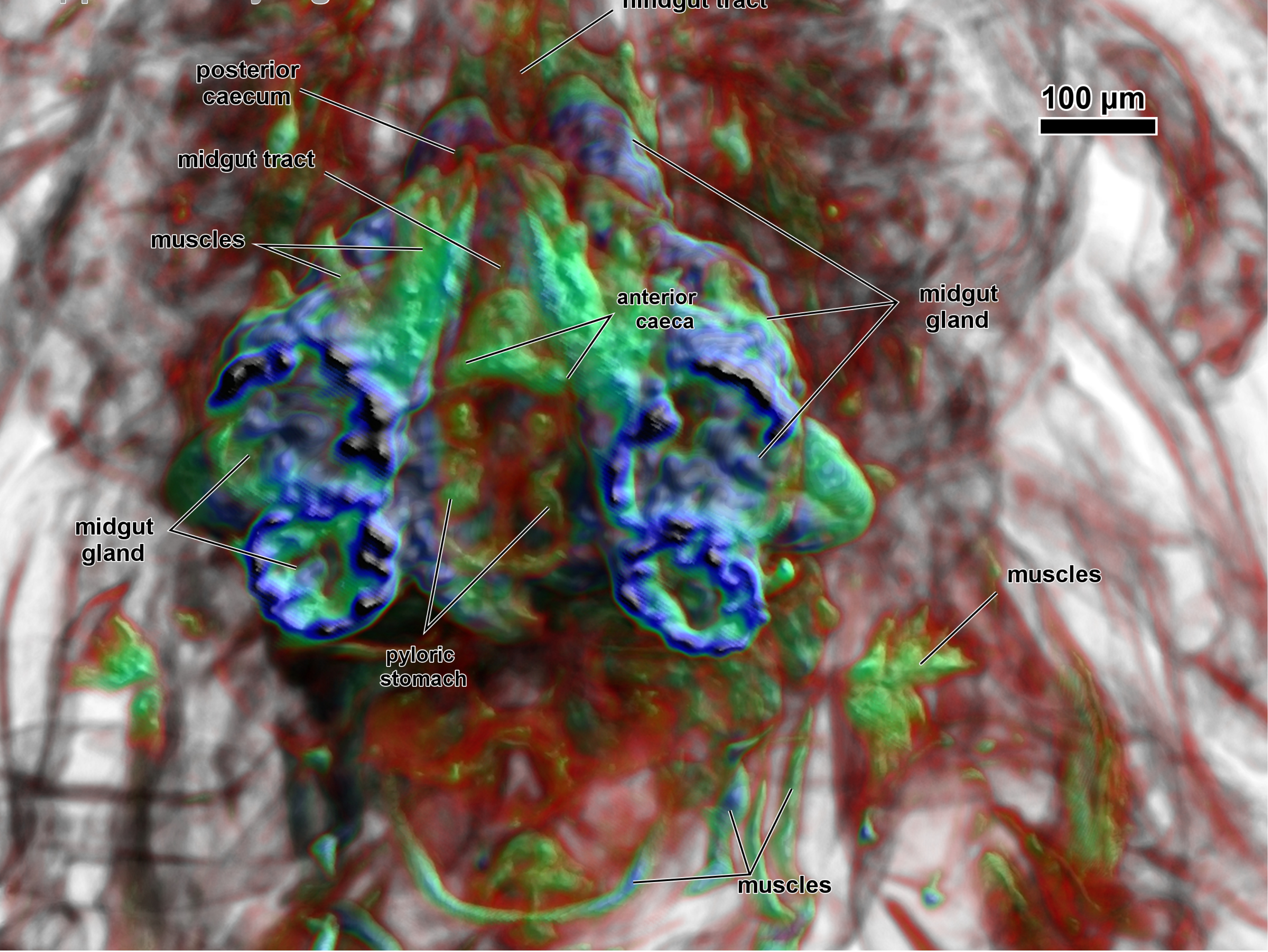

Supplementary Figure 14

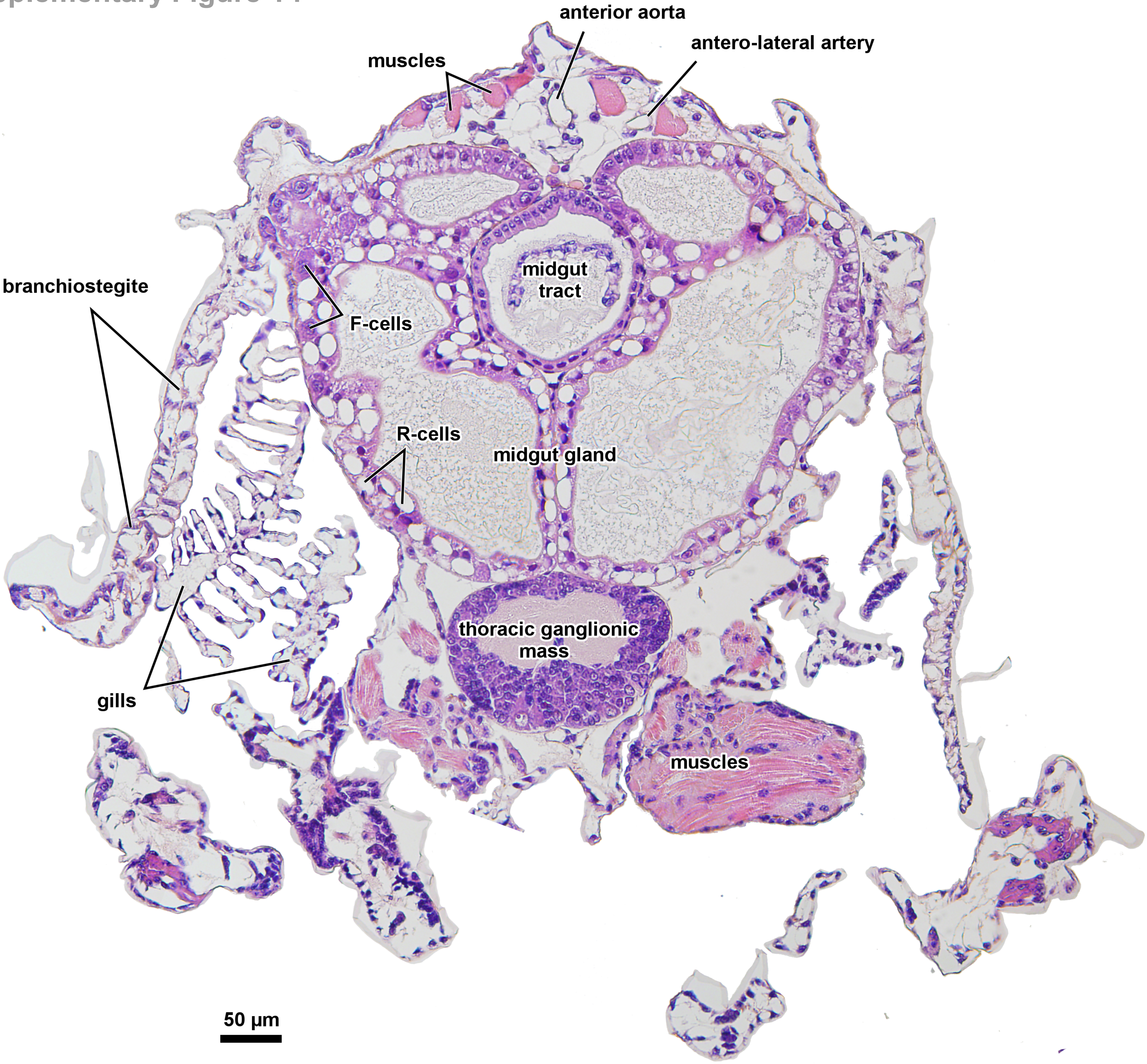

Supplementary Figure 15

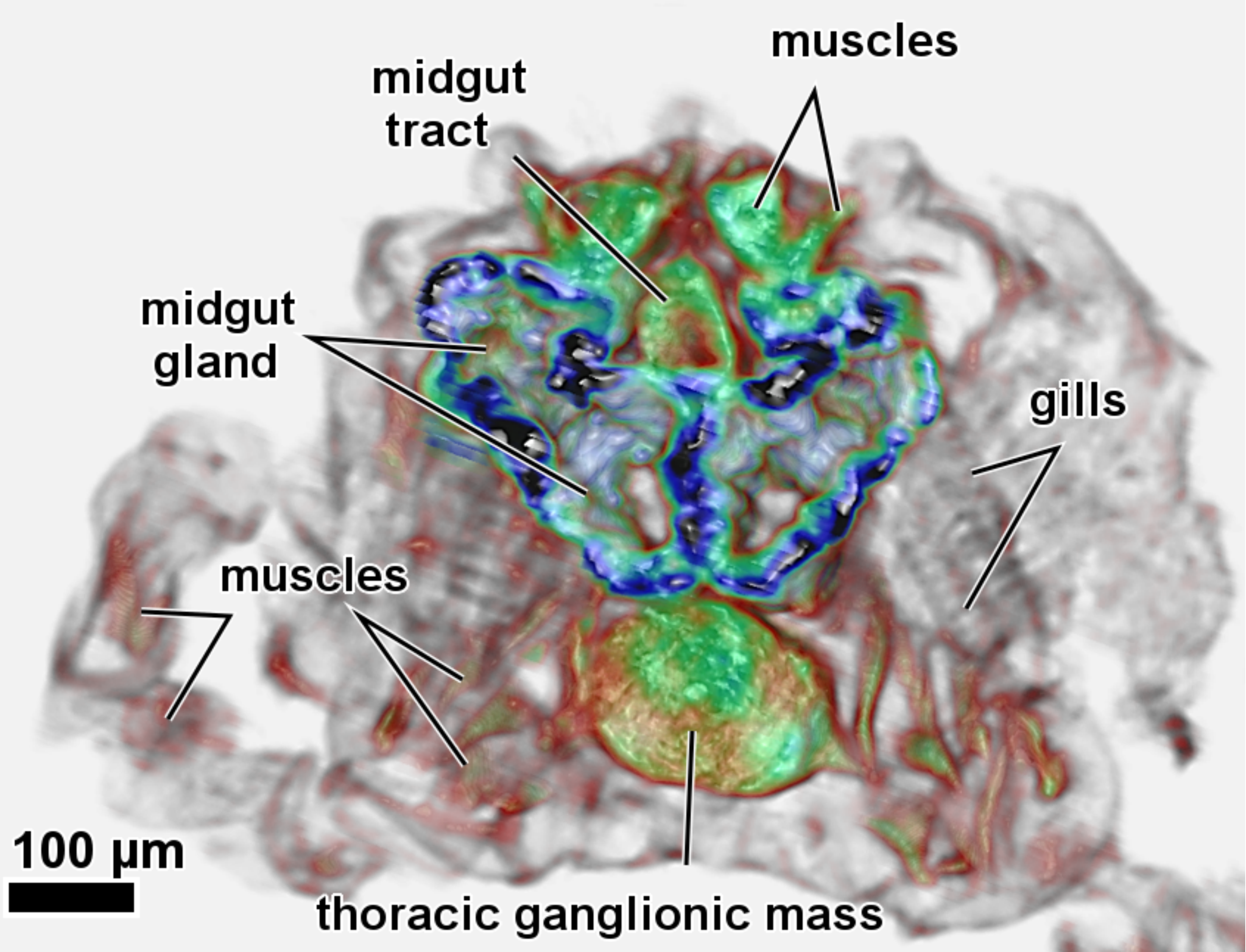

Supplementary Figure 16

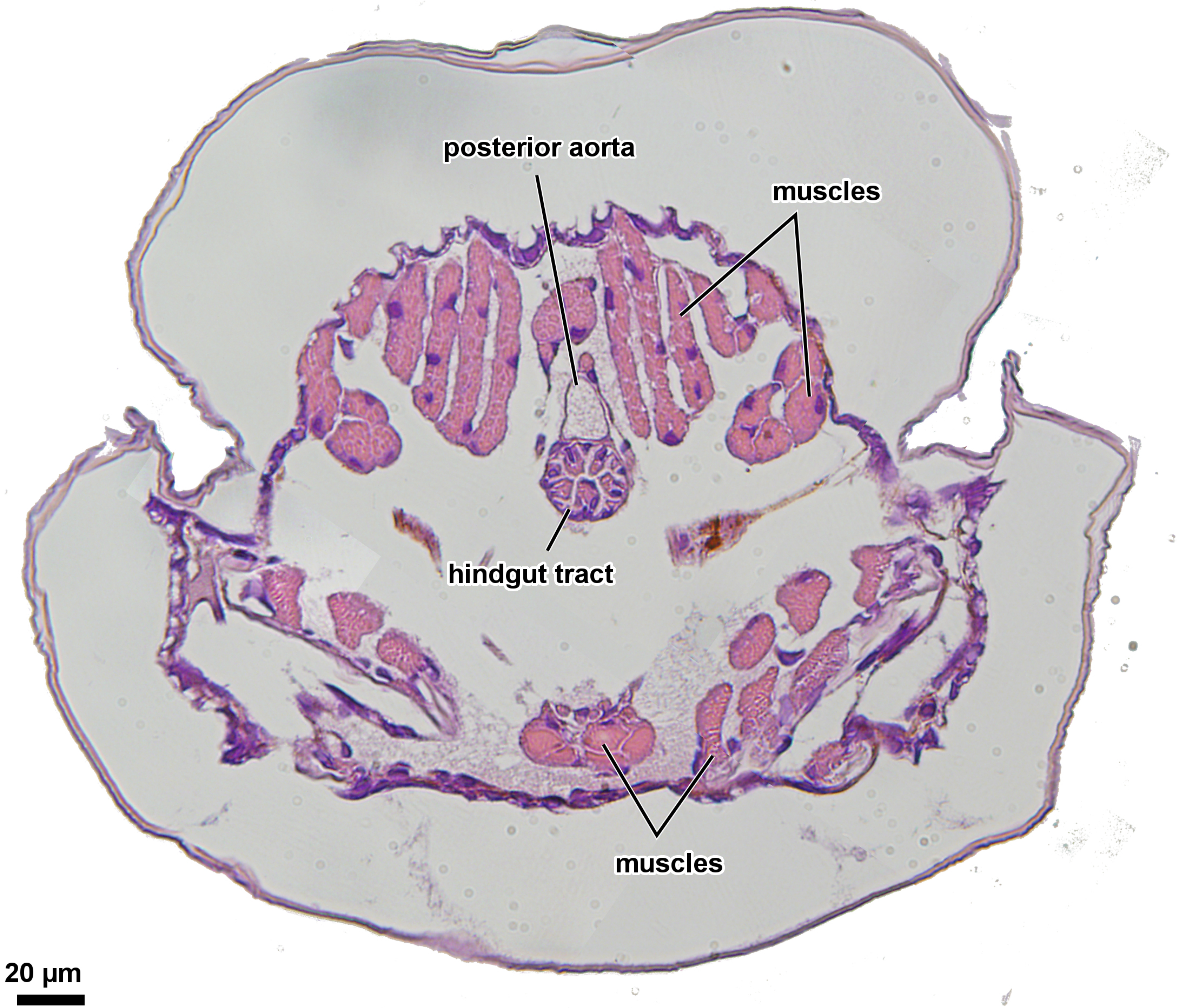

Supplementary Figure 17

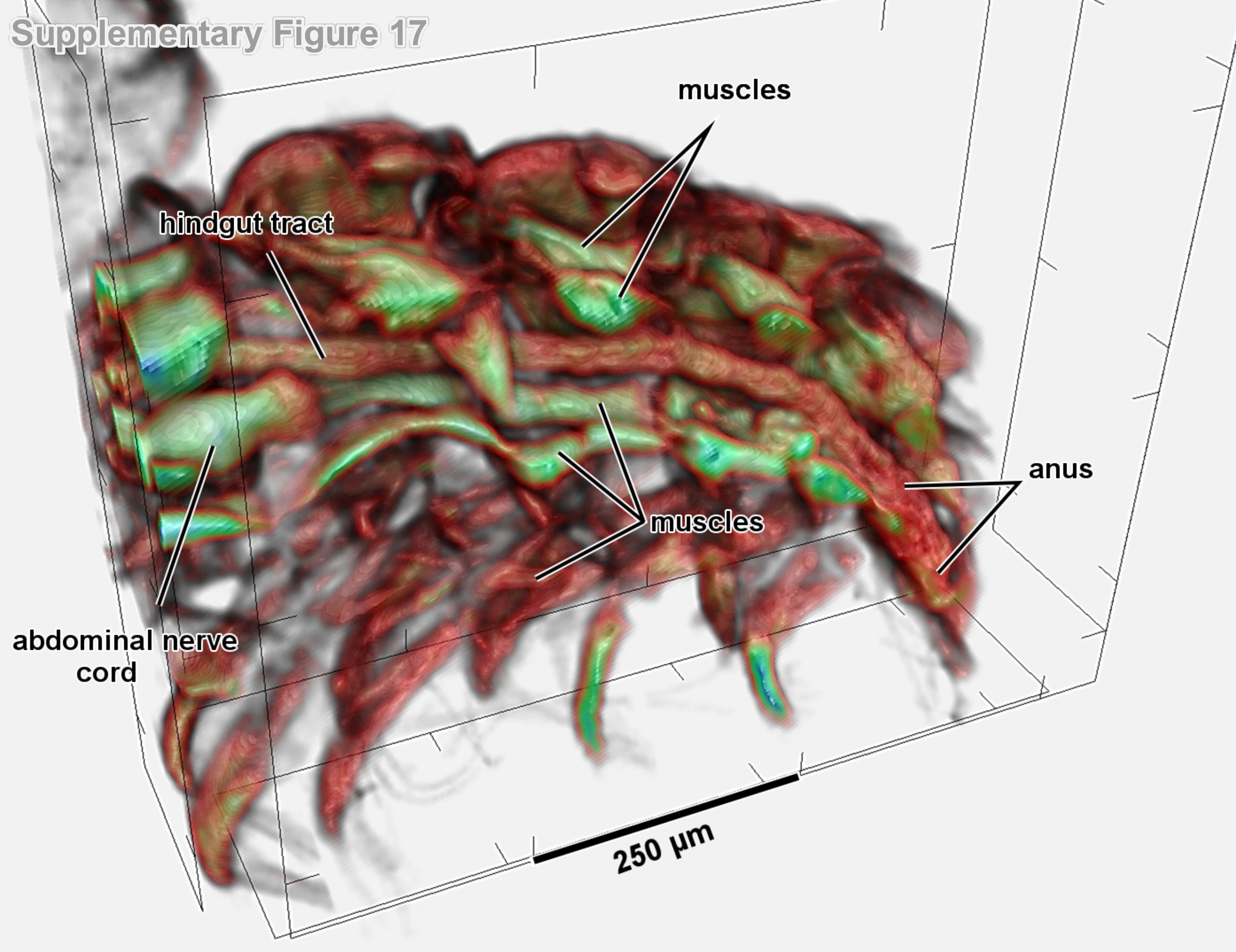

Supplementary Figure 18

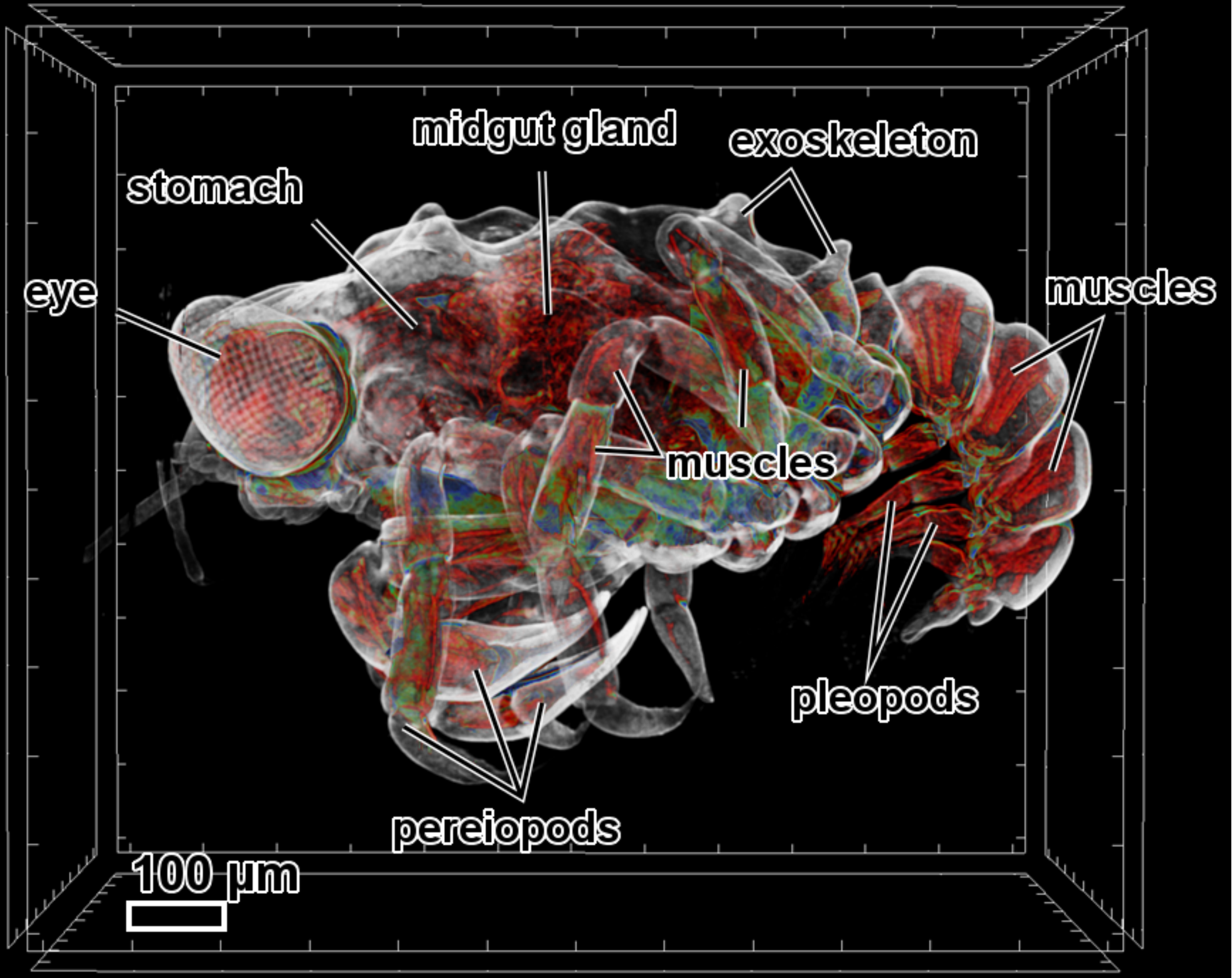

Supplementary Figure 19

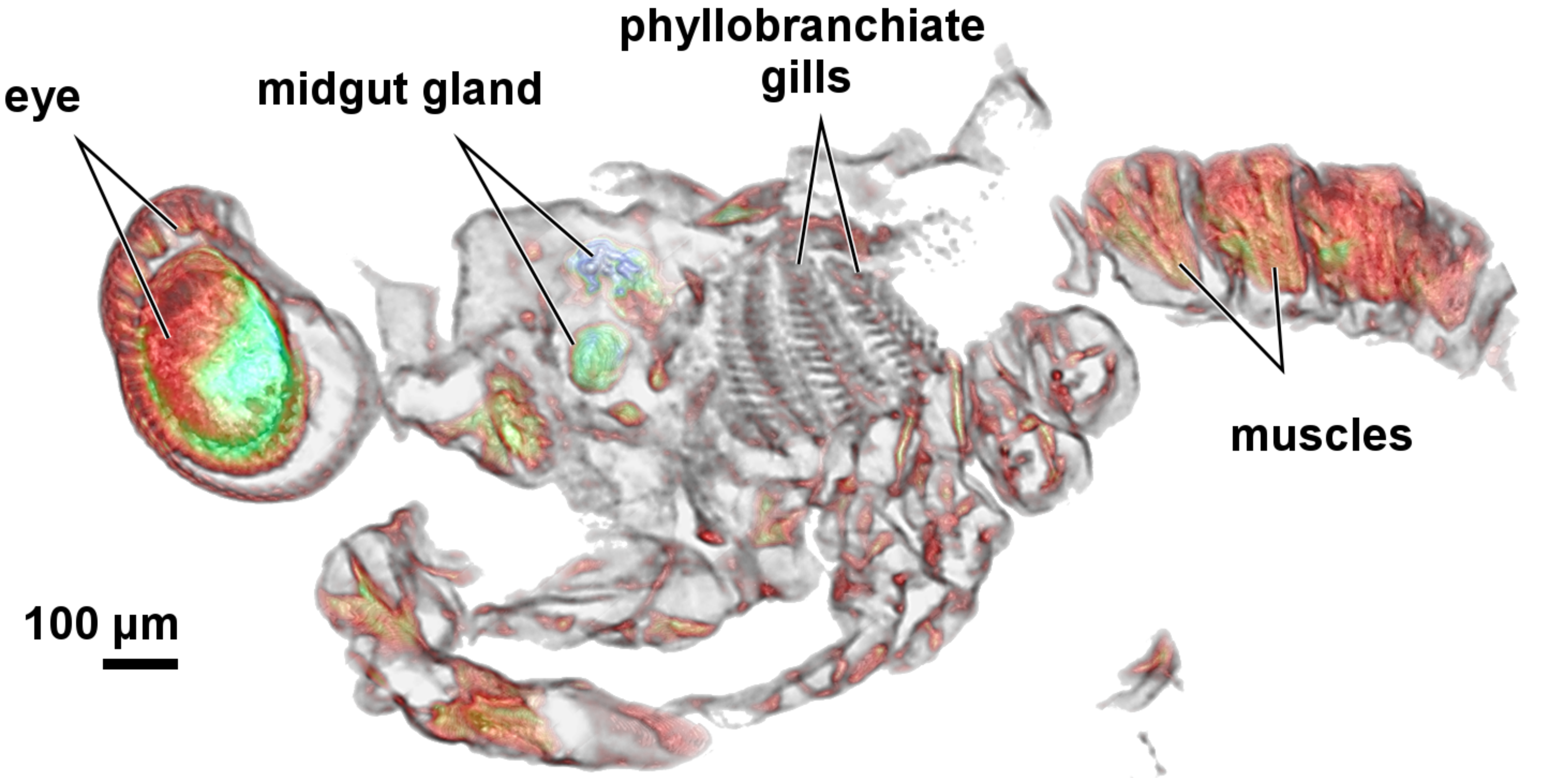

Supplement: Supplementary file 5 — Supplementary Information and Supplementary Figures [file 41598_2018_32709_MOESM5_ESM.pdf]
